# Theoretical and Experimental Studies of Molecular Interactions between Engineered Graphene and Phosphate Ions for Graphene-Based Phosphate Sensing

**DOI:** 10.1021/acsanm.3c04147

**Published:** 2023-12-18

**Authors:** Xue Yong, Thiba Nagaraja, Rajavel Krishnamoorthy, Ana Guanes, Suprem R. Das, Natalia Martsinovich

**Affiliations:** † Department of Chemistry, University of Sheffield, Sheffield S3 7HF, United Kingdom; ‡ Department of Industrial and Manufacturing Systems Engineering, Kansas State University, Manhattan, Kansas 66506, United States; § Department of Electrical and Computer Engineering, Kansas State University, Manhattan, Kansas 66506, United States

**Keywords:** oxygenated graphene, graphene-based sensors, phosphate sensors, curved graphene, graphene vacancy, electrical conductivity

## Abstract

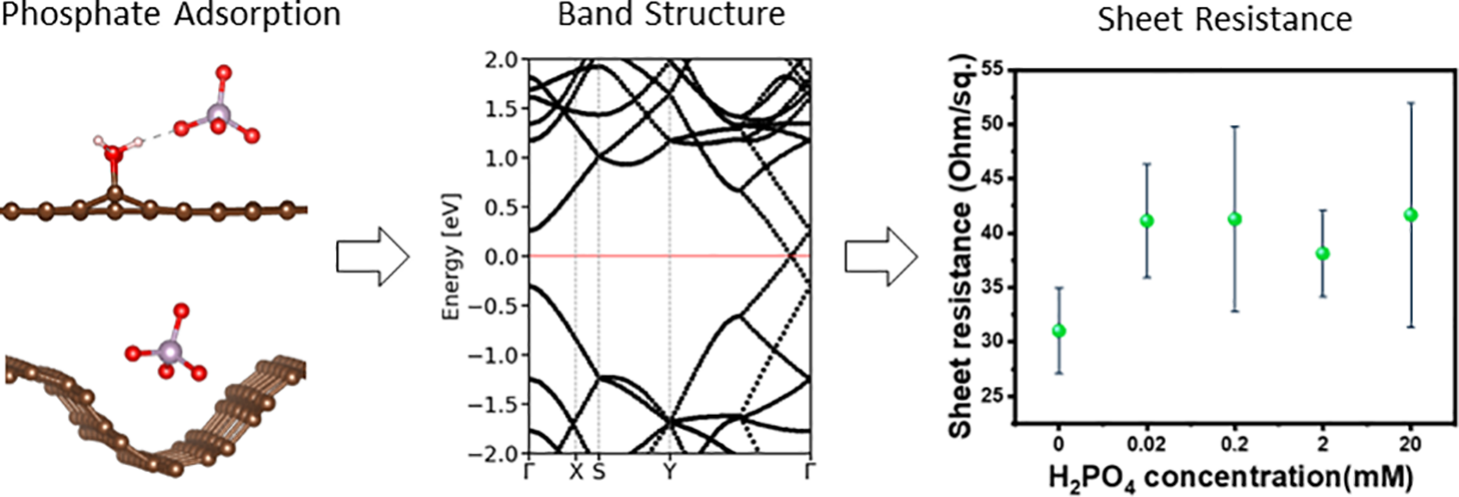

Fundamental understanding of the interactions of nanoscale materials with molecules of interest is essential for the development of electronic devices, such as sensors. In particular, structures and molecular interaction properties of engineered graphenes are still largely unexplored, despite these materials’ great potential to be used as molecular sensors. As an example of end user application, the detection of phosphorus in the form of phosphate in a soil environment is important for soil fertility and plant growth. However, due to the lack of an affordable technology, it is currently hard to measure the amount of phosphate directly in the soil; therefore, suitable sensor technologies need to be developed for phosphate sensors. In this work, pristine graphene and several modified graphene materials (oxygenated graphene, graphene with vacancies, and curved graphene) were studied as candidates for phosphate sensor materials using density functional theory (DFT) calculations. Our calculations showed that both pristine graphene and functionalized graphene were able to adsorb phosphate species strongly. In addition, these graphene nanomaterials showed selectivity of adsorption of phosphate with respect to nitrate, with stronger adsorption energies for phosphate. Furthermore, our calculations showed significant changes in electrical conductivities of pristine graphene and functionalized graphenes after phosphate species adsorption, in particular, on graphene with oxygen (hydroxyl and epoxide) functional groups. Experimental measurements of electrical resistivity of graphene before and after adsorption of dihydrogen phosphate showed an increase in resistivity upon adsorption of phosphate, consistent with the theoretical predictions. Our results recommend graphene and functionalized graphene-based nanomaterials as good candidates for the development of phosphate sensors.

## Introduction

Phosphorus is one of the essential nutrients for plant growth and crop production,^1,2^ where it aidsplant respiration and photosynthesis. Phosphorus is predominantly taken up by plants in the soluble form as orthophosphate (H_2_PO_4_^–^);^3^ thus, most phosphorus fertilizers, such as triple superphosphate, contain these phosphate species, although alternative fertilizers such as polyphosphates and phosphite are also used.^4^ Soils with phosphorus concentrations of 10—20 mg/kg are considered healthy and productive.^5^ Excess phosphorus in soil can be detrimental to plants and the environment because it can enter water bodies such as rivers, streams, lakes, and reservoirs and become one of the nutrient pollutants.^6,7^ Improved phosphorus management would promote the development of profitable and sustainable crop production while reducing water pollution. Monitoring and controlling the amount of phosphorus in soil and water require affordable and easy-to-use sensors.

Phosphorus is typically present in environments as phosphate, which is found in different protonation states, such as H_3_PO_4_ (pH < 2.2), H_2_PO_4_^–^ (2.2 < pH < 7.2), HPO_4_^2–^ (7.2 < pH < 12.4), and PO_4_^3–^ (pH > 12.4), depending on the pH of the environment.^8^ These species will be collectively referred to as “phosphate” in this paper. The pH of soil can range between 4.5 and 7.8,^9^ with pH values of 6–7 optimal for growing crops;^8^ therefore, phosphate is typically present in soil in the form of H_2_PO_4_^–^ and HPO_4_^2–^. The standard approaches for the detection of phosphate in soil and water rely on optical techniques, such as colorimetric methods, and electrochemical techniques, such as voltammetric and amperometric methods.^10^ However, colorimetric approaches suffer from interference from arsenates, silicates, sulfides, and oxidizing agents, while electrochemical phosphate sensors suffer from limited lifetimes. The effectiveness of these sensors needs to be further improved to achieve long-term stability, low maintenance, selectivity for phosphate, and sensitivity of measuring total phosphorus concentration down to at least 1 mg/L.^10^

Over the past few years, graphene-based nanomaterials have attracted great attention in the field of sensors because of their high surface-to-volume ratio, high carrier mobility, and electrical conductivity.^11−14^ Pristine graphene, with a single layer of *sp*^2^-hybridized carbon atoms, demonstrated 2630 m^2^/g theoretical specific surface area,^15^ with excellent thermal, mechanical, and electrical characteristics.^16^ Functionalized graphenes emerged as promising candidates for sensors because they possess more active sites for adsorption compared to pure graphene and have the potential to achieve selectivity through control of functional groups.^17,18^ In particular, graphene materials with oxygen functional groups have been used in gas sensors^19^ and humidity sensors.^20^ Theoretical calculations also suggested that defects, such as vacancies, can improve graphene’s sensing abilities by enhancing the adsorption of gas molecules^21^ and by modulating graphene’s electrical response properties.^22^ In addition to flat graphene sheets, three-dimensional graphene-based aerogels have been produced experimentally.^23^ Morphologically, graphene aerogels can be represented as curved graphene sheets.^24^ Experiments showed that graphene aerogels have high adsorption capacity for a variety of adsorbates, including organic molecules and inorganic ions.^23^ Computational modeling showed that curved graphene provided stronger adsorption of H_2_ and O_2_ compared to flat graphene^25^ and improved catalytic ability for hydrogen evolution.^26^ These studies suggest that curved graphene may also provide strong adsorption of phosphate and would be a useful sensor material. Overall, literature studies clearly show that pristine graphene and defect-containing, oxygen-functionalized, and curved graphene are effective sensor materials and can be good starting materials for the development of sensors for phosphorus in soil.

However, graphene-based phosphate sensors have not yet been reported. In a recent study, graphene-transition metal composites were used as adsorbents to remove phosphate from water;^27^ however, this approach relies on expensive and potentially toxic transition metals, therefore a cheaper functionalization such as oxygen, defects, or curvature control would be preferable. Another recent study investigated incorporation of phosphoric acid during synthesis of graphene oxide and showed that phosphate can bind to graphene oxide both by hydrogen bonding to graphene oxide’s functional groups and by covalent bonding under harsh synthesis conditions.^28^ This study supports functionalized graphene nanomaterials as good candidates for phosphate binding. Therefore, in this work, we investigated pristine graphene and engineered graphenes such as curved graphene, graphene with vacancies, and oxygenated graphene containing epoxide and hydroxyl groups as sensor materials for the detection of phosphate through density functional theory (DFT) calculations.

To identify suitable graphene-based materials for a phosphate sensor, we performed detailed DFT calculations to provide quantitative insight into how different phosphate species (PO_4_^3–^, HPO_4_^2–^, H_2_PO_4_^–^, and H_3_PO_4_) can be adsorbed on graphene and different modified graphene-based nanomaterials. The change in the electrical conductivities of graphene-based materials before and after the adsorption of phosphate was computed to reveal the effect of phosphate adsorption on the electrical conductivity of these graphene materials and to assess their ability as phosphate sensor. Finally, electrical sheet resistance measurements of graphene before and after phosphate adsorption were carried out to verify the predicted sensitivity of graphene toward phosphate.

## Computational Details

All density functional theory (DFT) calculations were performed using the Vienna *ab initio* simulation package (VASP).^29−31^ The nonlocal van der Waals density functional (vdW-DF2) of Lee et al.^32^ and the generalized gradient approximation (GGA) were used to describe the exchange-correlation energy. This functional was chosen because of its good accuracy in describing physisorption and binding in molecular and layered solids^33,34^ and was used without modification, e.g., without change to the recommended value of the parameter *Z*_AB_ = −1.8867.^32^ To describe the expansion of the electronic eigenfunctions, the projector-augmented wave (PAW) method was applied with the kinetic energy cutoff of 700 eV. Positions of all atoms were fully relaxed, until the changes in the total energy and force were below 10^–6^ and 0.02 eV Å^–1^, respectively. The Brillouin zone was sampled with a 4 × 4 × 1 k-point grid of the Monkhorst–Pack scheme in all calculations. A 20 Å vacuum was set above the slabs to avoid interaction between the periodic images. For comparison, we also considered effect of solvation on phosphate adsorption, using calculations with implicit water solvent using VASPsol.^35,36^

**Figure 1:**
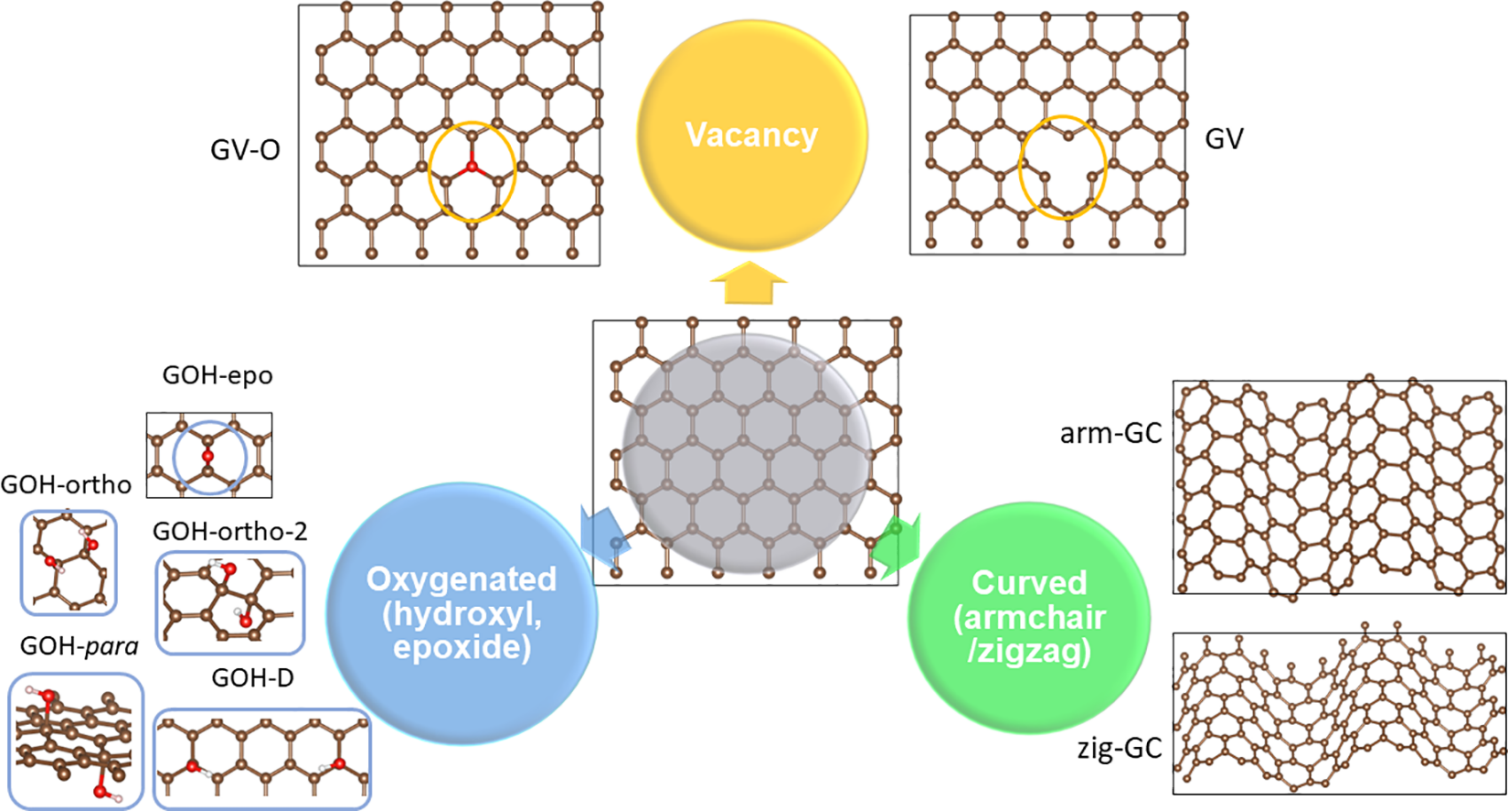
Structural models of the studied graphene, oxygenated graphene (GO, GOH), graphene with a carbon monovacancy (GV) and oxygen-filled vacancy (GV-O), and curved graphene (GC). Carbon atoms are shown in brown, oxygen atoms in red, and hydrogen atoms in light gray. The specific details of the structures are discussed in the text.

We modeled pristine graphene (G), oxygenated graphene, graphene with a carbon monovacancy, and curved graphene (Figure 1). For all models, we used the 6 × 6 × 1 rectangular graphene supercell (72 C atoms) shown in Figure 1. Our tests of adsorption energies of PO_4_^3–^ on pristine graphene in 4 × 4 × 1, 5 × 5 × 1. and 6 × 6 × 1 supercells showed that the 6 × 6 × 1 size gives converged adsorption energy values. While it has been recently reported that the trends in adsorbate/substrate binding energies vs substrate sizes calculated using nonlocal functionals differed from higher-level methods such as diffusion Monte Carlo (DMC),^37^ we believe that keeping the substrate (surface supercell) size constant in our studies would make these size effects consistent in all of our surface/adsorbate systems, and therefore, the trends in adsorption energies of different phosphate species on differently functionalized substrates should be valid.

We considered several types of GO: graphene with one epoxide functional group (GO-epo) and four configurations of graphene with a pair of hydroxyl groups: GOH-ortho (two hydroxyls next to each other, on the same side of the graphene sheet), GOH-ortho-2 (two hydroxyls next to each other, on the opposite sides of the graphene sheet), GOH-para (two hydroxyls in the para-position relative to each other, on the opposite sides of the graphene sheet), and GOH-D (two hydroxyls separated by the distance of three lattice vectors of graphene, on the same side of the graphene sheet), all shown in Figure 1. These simple model structures correspond to the low oxygen concentration range of realistic graphene oxides.^38^ Previous experimental and computational studies of graphene oxide and reduced graphene oxide showed that oxygen functional groups are likely to be randomly arranged in clusters.^38−40^ The structures GOH-ortho and GOH-ortho-2 are examples of small clusters of hydroxyls, while the structures GOH-para and GOH-D are examples of random distribution of functional groups or groups belonging to separate neighboring clusters. Structure GOH-D, in particular, was chosen because this arrangement of hydroxyl groups was able to form two simultaneous hydrogen bonds with phosphate and therefore enabled us to investigate the relationship between the strength of binding and electronic response of graphene.

For graphene with a vacancy, we considered a single vacancy (GV) and a vacancy filled with a substitutional three-coordinated O atom (GV-O). For curved graphene, we considered a graphene sheet curved along the armchair or zigzag direction (arm-GC and zig-GC), which was obtained by compressing the graphene lattice in the armchair or zigzag direction, respectively, displacing the atoms vertically along a sinusoidal wave in the compression direction and optimizing the structure.

Electronic band structures were unfolded using the algorithm embedded in VASP.^41^ Electrical conductivity σ was computed using Boltzmann transport equation with constant relaxation time as implemented in Boltztrap2 and pymatgyn,^42,43^ using the formula
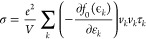
where  is the Fermi–Dirac distribution function,  means summation over *k*-points along the specified path in the Brillouin zone, *e* is the electron charge, *V* is the volume, *v*_*k*_ is the group velocity, *τ*_*k*_ is the relaxation time, *ε*_F_ is the Fermi energy, *k*_B_ is the Boltzmann constant, and *T* is the temperature. The temperature of 300 K was used, while *τ*_*k*_ was set as constant at 1 × 10^–14^ s. Constant relaxation time was used, because it has been shown to give reasonable agreement with experiment for room temperature studies.^44−46^ The volume was taken as the surface area of graphene multiplied by the thickness of the single layer graphene, where the thickness is estimated to be 3.5 Å.

## Experimental Details

Graphene material was synthesized via liquid phase exfoliation of graphite using the protocol presented in a recently published paper,^47^ based on the procedure proposed in an earlier study.^48^ Liquid phase exfoliation (LPE) was chosen because it enabled us to synthesize an intermediate platform where one can synthesize a large number of pure graphene flakes. Use of graphene from the LPE technique also satisfies practical considerations for fabrication of sensors, such as low cost and scalability of graphene production. To obtain graphene inks, graphene processed via liquid phase exfoliation of graphite with an ethyl cellulose surfactant was dispersed into cyclohexanol and terpineol (70 mg/mL concentration). Here, 50 μL of the prepared ink was drop cast on to a 125-μm thick polyimide film and heat treated at 100 °C for 30 min. Subsequently, the graphene film was annealed in an air atmosphere at 350 °C for 2 h to remove ethyl cellulose polymers to get a pure graphene film. The highly optimized process presented in ref (47) enables us to produce few layer graphene (average thickness ≤10 layers). The Supporting Information in ref (47) presents the details on the characterization of the inks, validating their quality. We believe that the trends in electrical conductivity of this few-layer graphene should not be substantially different from those of single-layer graphene.

Potassium dihydrogen phosphate (KH_2_PO_4_, ≥99.0% Sigma-Aldrich) was used as a representative phosphate species, where different concentrations of it (0.02, 0.2, 2, and 20 mM) were drop coated (50 μL each) on the graphene film and then air-dried. A four-probe electrical resistivity measurement was chosen because such a setup provides true resistivity (and hence the sheet resistance) of sensor electrodes by eliminating any associated contact resistance. The sheet resistance of all four samples was measured by using a S-302 four-point probe measurement unit interfaced with a Keithely 4200 source measure unit. A similarly prepared but bare graphene film (without any phosphates) was used as a standard for comparison. To ensure the reliability and reproducibility of the experimental results, measurements carried out on at least five samples were used to estimate the average sheet resistance.

## Results

### Geometries of Graphene-Based Materials

All of the graphene, oxygenated graphene, and graphene with vacancy structures were planar (Figure 1). C–C bond lengths involving carbon atoms bonded to oxygens in GV-O, GO-epo, and GOH structures were slightly extended to 1.47–1.58 Å, compared to the ideal 1.42 Å C–C bond distance in graphene. The C–C bonds in curved graphene were very similar to those in pristine graphene and varied between 1.41–1.43 Å both in arm-GC and zig-GC. However, due to the curvature of the GC sheets, some of the second neighbor C–C distances decreased from 2.46 Å in pure graphene up to 2.41 Å. These slight changes in interatomic distances suggest that functionalization, vacancies, and curvature will alter the electronic structure of graphene and may facilitate the adsorption of phosphate.

### Adsorption of Phosphate Species: PO_4_^3–^

**Figure 2:**
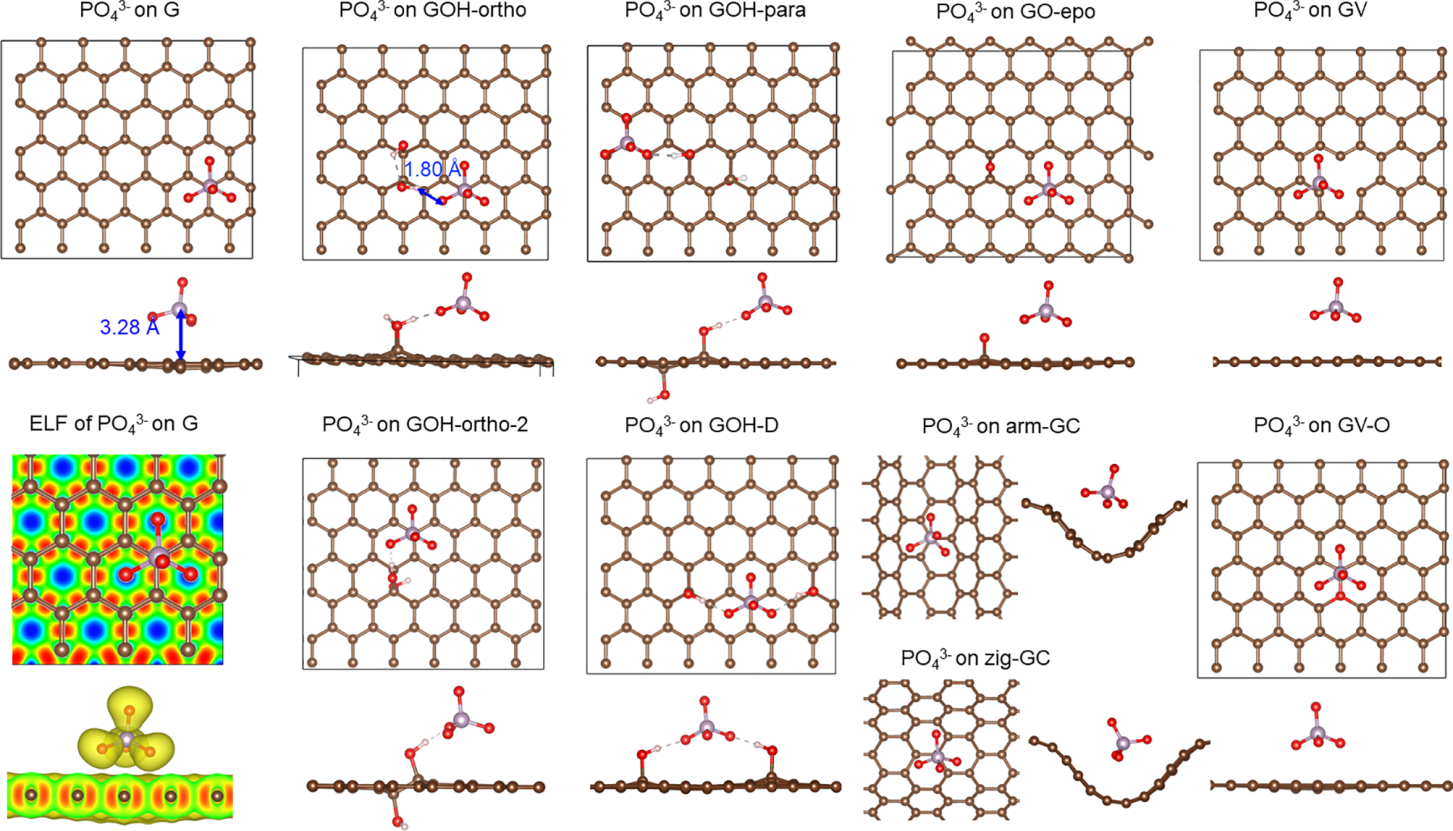
Most stable adsorption configurations for PO_4_^3–^ on pristine graphene (G), hydroxyl- (GOH) and epoxide-containing (GO-epo) graphene, curved graphene (arm-GC and zig-GC), and graphene with vacancy (GV) and with oxygen-filled vacancy (GV-O). Top and side views are shown for all systems. Electron localization function (ELF) is shown for PO_4_^3–^ on pristine graphene: a slice through the graphene plane and a view in the vertical plane. The color scheme in the ELF plot ranges from yellow (highest electron localization, ELF value of 0.46) through red to blue, which means a lack of electrons. In the ball and stick structures, carbon atoms are shown in brown, oxygen atoms in red, and phosphorus atoms in light purple.

**Figure 3:**
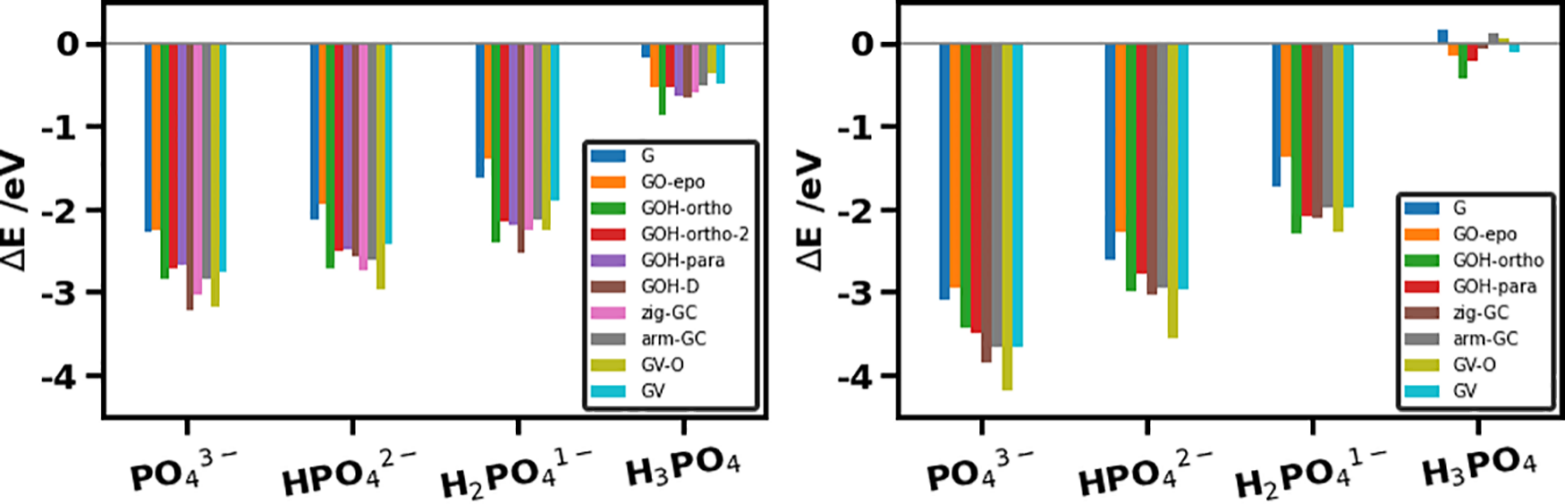
Adsorption energies for phosphate species (PO_4_^3–^, HPO_4_^2–^, H_2_PO_4_^–^, H_3_PO_4_) on graphene-based materials: (left) in a vacuum and (right) in implicit water solvent.

To find the most stable adsorption configurations of phosphate on graphene, different positions of phosphate were considered, and the structures with the most negative total energies were identified. Only the most stable structures for each phosphate on each graphene surface were used for further analysis. First, the preferred adsorption site for PO_4_^3–^ on pure graphene was found, by placing the PO_4_^3–^ species either at the top site above a C atom or at the ring center site of pristine graphene. The position above a C atom was more stable; therefore, this position was then used as a starting point for phosphate adsorption on functionalized graphenes. Placements of PO_4_^3–^ above C at different distances from the defects or functional groups were explored, and the most stable position for of PO_4_^3–^ on each functionalized graphene was identified. For curved graphene, placements of PO_4_^3–^ at the top or in the valley of the curve with different numbers of O pointing down were considered. The positions that were found stable for PO_4_^3–^ were then used for hydrogenated phosphates, where we additionally investigated the possibility of the phosphates’ OH groups pointing either up or toward the surface. For each of the adsorbate species on each graphene surface, the adsorbed structure with the most negative energy was selected, and these structures were used to compute adsorption energies and for all further analyses. The adsorption energies were computed as Δ*E* = *E*_*AB*_ – *E*_*A*_ – *E*_*B*_, where *E*_*AB*_ is the total DFT energy of a phosphate species adsorbed on graphene, and *E*_*A*_ and *E*_*B*_ are the total DFT energies of graphene with/without functionalization and of the isolated phosphate species, respectively. The most stable adsorption structures are shown in Figure 2 for PO_4_^3–^ and Figures S1–S3 for HPO_4_^2–^, H_2_PO_4_^–^, and H_3_PO_4_, and the adsorption energy values for all adsorbates are tabulated in Table S1 and are presented in Figure 3.

The preferred adsorption configuration of the PO_4_^3–^ ion on pure graphene had the P atom above the C atom, with the C–P distance of 3.28 Å (Figure 2), with three O atoms of phosphate pointing to the centers of the nearest six-membered rings of the graphene and the fourth O atom pointing away from the graphene slab. No covalent bonds were formed between the adsorbate and graphene. However, as shown in Figure 3, pristine graphene provided strong adsorption of PO_4_^3–^ with an adsorption energy of −2.28 eV. To clarify the nature of the interaction, electron localization function (ELF) was calculated to describe the bonding character in the surface–adsorbate system. Figure 2 shows regions of electron localization (shown in yellow and red) between pairs of C–C atoms and electron deficient regions (in blue) at the centers of the six-member rings of graphene. No electron localization was seen between carbon and oxygen atoms, proving that no surface–adsorbate chemical bonds were formed. Three oxygens of PO_4_^3–^ pointed to the centers of the six-membered rings, suggesting electrostatic interactions between the electronegative oxygens and the electron-deficient regions of graphene.

**Figure 4:**
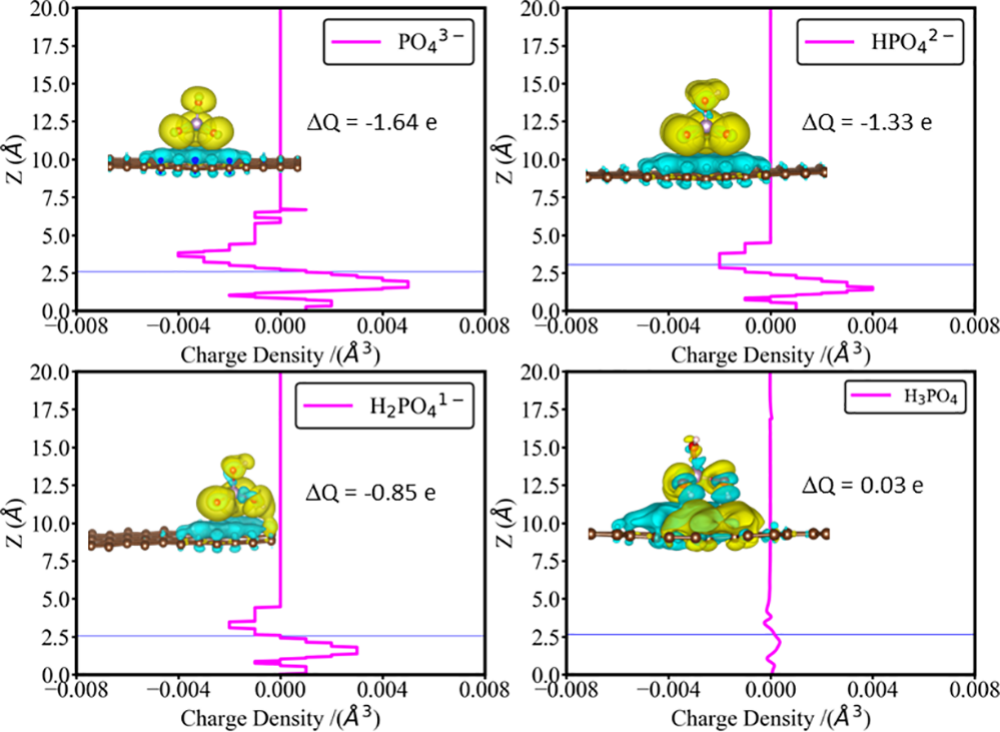
Charge density difference plots for phosphate species (PO_4_^3–^, HPO_4_^2–^, H_2_PO_4_^–^, and H_3_PO_4_) adsorbed on pure graphene. The charge density differences were calculated as the differences of electron densities of the combined graphene+phosphate system minus the isolated graphene and phosphate. Blue bubbles show the gain of electron density, while yellow bubbles show the loss of electron density. The pink lines show height-resolved charge density differences averaged across horizontal planes: positive values indicate electron gain at this particular value of *z*, while negative values indicate electron loss. The graphene surface is at *z* = 0, while the thin blue horizontal line shows the position of the P atom of the adsorbate. The amount of charge transferred between the adsorbate and substrate (Δ*Q*) is shown as insets in the figures. Negative values of Δ*Q* indicate electron transfer from the adsorbate to graphene, while positive values of Δ*Q* indicate electron transfer from graphene to adsorbate.

To obtain further understanding of the nature of the interaction between graphene and adsorbed phosphate, the charge density difference between the combined graphene+phosphate system and the isolated graphene and phosphate was analyzed. The isocontours in Figure 4 show electron accumulation as blue bubbles and electron depletion as yellow bubbles. These electron density difference data show electron transfer from PO_4_^3–^ to graphene. Bader charge analysis shows that the charge on the adsorbed PO_4_^3–^ species is −1.36 e, which is less negative than the formal charge of this ion; therefore, −1.64 electrons have been transferred from the phosphate to graphene. These results suggest that PO_4_^3–^ adsorbs on graphene thanks to charge transfer and electrostatic interactions of the negatively charged phosphate ion and the polarizable graphene substrate.

PO_4_^3–^ adsorbed on all graphene-based materials in a similar manner, with the P atom above a C atom, and O atoms pointing to the centers of six-membered rings (Figure 2). In particular, when a vacancy or an oxygen-filled vacancy was present, phosphate preferred to adsorb immediately next to the vacancy. When an epoxide was present, phosphate adsorbed one lattice distance from the epoxide, suggesting some repulsion between the phosphate and the epoxide. On curved graphene, PO_4_^3–^ preferred to sit in the valley of the curved structures. When hydroxyl was present, the most stable adsorbed PO_4_^3–^ structures similarly adsorbed with three O pointing down and additionally formed a hydrogen bond with the −OH groups of GOH.

With the exception of GO-epo, which showed very slightly weaker adsorption of PO_4_^3–^ than on pure graphene, all modified graphene materials exhibited enhanced adsorption of PO_4_^3–^ ions, with *E*_ads_ ranging from −2.69 to −3.23 eV, i.e., up to 0.95 eV stronger adsorption compared to pure graphene. The stronger adsorption on GOH structures is explained by the hydrogen bond formed between PO_4_^3–^ and the surface hydroxyl. To explore the effect of the arrangement of surface hydroxyls, graphenes with a pair of hydroxyls on the same side or opposite sides of the graphene sheet were compared (GOH-ortho and GOH-ortho-2, respectively). In both cases, the phosphate ion formed only one hydrogen bond with one hydroxyl; even when another nearby hydroxyl was available in GOH-ortho, a second hydrogen bond was not formed, indicating that the hydroxyl’s orientation was not favorable for hydrogen bonding. Thus, the possible energy gain due to the formation of a second hydrogen bond was not sufficient to change the orientation of the surface hydroxyls or of the adsorbed PO_4_^3–^. The adsorption of PO_4_^3–^ on GOH was slightly stronger when both adsorbates were on the same side of the graphene sheet and slightly stronger when the hydroxyls were close together (GOH-ortho) than further apart (GOH-para). To investigate an arrangement where multiple surface–adsorbate hydrogen bonds were possible, we designed the structure GOH-D (Figure 1), where two surface hydroxyls were placed three lattice vectors apart from each other and were suitably positioned to form two hydrogen bonds with the adsorbed PO_4_^3–^ ion. This structure provided the most negative adsorption energy (−3.23 eV). According to previous experimental and computational studies of graphene oxide and reduced graphene oxide, oxygen functional groups are likely to be randomly arranged in clusters.^38−40^ Therefore, a variety of hydroxyl arrangements is likely to exist in realistic materials, and some of these hydroxyl arrangements may form multiple hydrogen bonds with phosphates and therefore trap adsorbed phosphate strongly.

The adsorption of PO_4_^3–^ on graphene containing a vacancy or a filled vacancy was also stronger than on pure graphene, which suggests that defective graphene has a good sorption ability for phosphate. The enhanced adsorption can be attributed to the interaction of the adsorbed phosphate with the lone pairs of carbon atoms of the vacancy or with the lone pair of the substitutional oxygen. Similarly, strong adsorption of PO_4_^3–^ was found on the curved graphene. This can be attributed to stronger dispersion interactions: since the phosphate ion prefers to sit in the valley of the curved graphene structures, it is able to interact with more carbon atoms compared to the case of pure graphene. In summary, both functionalized graphene (oxygenated or vacancy containing) and nonplanar graphene provide strong adsorption of phosphate.

### Adsorption of HPO_4_^2–^, H_2_PO_4_^–^, and H_3_PO_4_

The hydrogenated phosphate species (HPO_4_^2–^ and H_2_PO_4_^–^ ions and phosphoric acid H_3_PO_4_) adsorbed on graphene and functionalized graphenes in a manner very similar to that of PO_4_^3–^, with the phosphorus atom above a carbon atom. For the monohydrogenated HPO_4_^2–^ ion, the preferred adsorption configurations had the hydroxyl group pointing upward, away from the surface, with three oxygen atoms pointing toward the surface, same as for the adsorbed PO_4_^3–^. For the dihydrogenated H_2_PO_4_^–^ ions and trihydrogenated H_3_PO_4_ molecule, the preferred adsorption configurations similarly had as few as possible hydroxyl groups pointing toward the surface. These preferred adsorption configurations again confirm the key role of the interaction of oxygen atoms of the phosphate species with the electron-poor regions in the centers of graphene’s six-membered rings.

To explore the nature of the interaction more quantitatively, electron density difference plots and Bader charges for the hydrogenated phosphates adsorbed on pure graphene were analyzed (Figure 4). Similar to adsorbed PO_4_^3–^, significant charge transfer was observed for HPO_4_^2–^ and H_2_PO_4_^–^ ions adsorbed on graphene. The height-resolved line plots, which show charge distribution perpendicular to the surface (calculated as electron density differences averaged across horizontal planes), reveal that the amount of charge transfer was larger for the adsorbates with higher ionic charges. This is confirmed by Bader charge analysis, which showed that adsorbed HPO_4_^2–^ and H_2_PO_4_^–^ donated −1.33 and −0.84 e, respectively, to graphene. In contrast, the electron transfer pattern of the adsorbed H_3_PO_4_ molecule was more complex, showing a redistribution of charge both within the adsorbate and within the graphene substrate rather than directional surface–adsorbate charge transfer. This lack of directional charge transfer is confirmed by the height-resolved electron density difference plot: the amount of charge transferred, averaged across horizontal planes, was less than 0.001 e/Å^3^. Bader charge analysis similarly showed that only 0.03 e was transferred from graphene to H_3_PO_4_; i.e., the direction of charge transfer was the opposite from the case of the adsorbed ions, but the amount of charge transfer was negligible, probably because of effective delocalization of electrons over graphene’s π-system. This different charge transfer pattern also explained why the adsorption of PO_4_^3–^, HPO_4_^2–^, and H_2_PO_4_^–^ was stronger than that of H_3_PO_4_, because the latter lacked additional stabilization given by the surface–adsorbate charge transfer.

The adsorption of HPO_4_^2–^ and H_2_PO_4_^–^ ions and phosphoric acid H_3_PO_4_ on graphene and all functionalized graphene substrates was weaker compared to the adsorption of PO_4_^3–^: the range of adsorption energies for HPO_4_^2–^ was −1.95 to −2.97 eV. For H_2_PO_4_^–^, the range was −1.40 to −2.54 eV, and for H_3_PO_4_, the range was −0.18 to −0.87 eV. Thus, adsorption was progressively weaker as the ionic charge decreased, consistent with the decreasing amounts of electron transfer for the low-charge and neutral adsorbates.

### Adsorption in the Implicit Solvent Environment

The calculations presented in the previous section considered the graphene substrate and the adsorbates in the vacuum environment. However, the environment relevant for a phosphate sensor is the water environment. To include the effect of the water environment in an approximate manner, we modeled the same adsorption configurations in implicit water solvent and calculated their adsorption energies relative to solvated graphene and phosphate. The adsorption energies obtained in implicit water solvent are presented in Table S2 and summarized in the right panel of Figure 3. The key trends in these adsorption energies are the same as those in the vacuum environment: the adsorption of phosphate ions on modified graphenes (except GO-epo) is always stronger than that on pristine graphene. The adsorption of the highly charged ions is stronger than the adsorption of the low-charge ions and especially of neutral phosphoric acid.

Unlike the vacuum environment where the adsorption energies of phosphoric acid were negative values between −0.18 and −0.87 eV, in the water environment, the adsorption of phosphoric acid was weaker, with adsorption energies closer to zero (the small positive values of 0.06–0.15 eV obtained for phosphoric acid on G, arm-GC, and GV-O are likely due to the limited accuracy of the calculations). These results suggest that phosphoric acid is stabilized in the water environment and has little or no enthalpic driving force for adsorption; additionally, the entropic factor would favor phosphoric acid remaining in solution. Therefore, graphene materials would not be suitable sensor materials to detect phosphoric acid. In contrast, adsorption of ions, especially PO_4_^3–^ and HPO_4_^2–^, was noticeably stronger in the water environment than in vacuum, which suggests that isolated phosphate ions are not as effectively stabilized in the water environment.

It must be noted that the implicit solvent model does not include the possibility of hydrogen bonds forming between phosphate species and water molecules. Hydrogen bonds are likely to stabilize species in solution more than adsorbed species because solutes have all their O or OH groups available for hydrogen bonding, while adsorbed species have some of these groups engaged in binding to the surface. However, this is likely to affect all phosphate adsorbates equally; therefore, the key trend of modified graphenes being better for adsorption of phosphates than pristine graphene is likely to remain true.

Furthermore, the description of the solvent environment through the implicit solvent model describes only the enthalpic factors but does not include vibrational and configurational entropy terms. Presence of explicit solvent would include the entropic driving force as well as adsorption and desorption of phosphate and competition with water molecules for adsorption sites on the graphene surface; however, this would be computationally unfeasible in the quantum chemistry framework.

### Selectivity of Adsorption of Phosphates vs NO_3_^–^

While the strength of adsorption can be seen as a measure of sensitivity of the sensor material (in this case, graphene materials) to the adsorbed species, another important consideration for sensors is their selectivity toward the species of interest. Nitrate ions are commonly present in soil and water; therefore, we studied the selectivity of adsorption of phosphates vs NO_3_^–^. The results presented above for the adsorption of phosphates on different graphene compounds led us to conclude that the hydrogen bonding interaction between surface hydroxyls and phosphate adsorbates is one of the key factors responsible for strong adsorption. Therefore, to study the selectivity of adsorption of phosphates vs NO_3_^–^, we chose pristine graphene and the GOH-D structure which enabled the strongest adsorption of PO_4_^3–^ (−3.23 eV) by forming two hydrogen bonds with the phosphate species.

**Figure 5:**
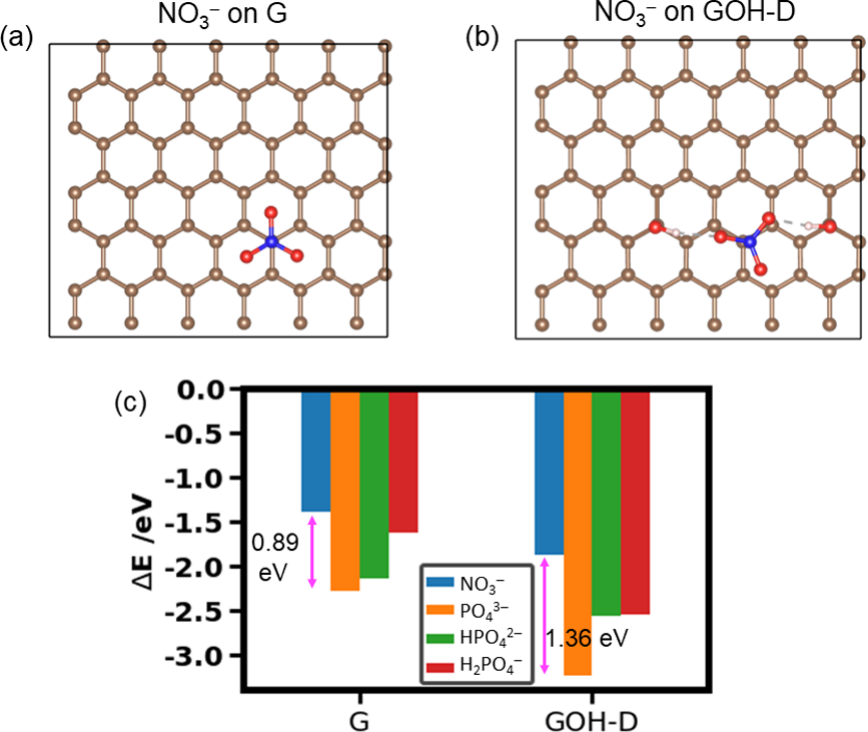
Adsorption selectivity of phosphates vs NO_3_^–^: adsorption configurations of NO_3_^–^ (a) on G and (b) on GOH-D and (c) adsorption energies for NO_3_^–^, PO_4_^3–^, HPO_4_^2–^, and H_2_PO_4_^–^ on G and GOH-D.

The NO_3_^–^ ion adsorbed in a way similar to that of the PO_4_^3–^ ion, despite the difference in the geometries of the planar nitrate ion and the pyramidal phosphate ion. On pristine graphene (G), the nitrate adsorbed with its N atom above a C atom and with the three oxygens of nitrate pointing toward the centers of graphene’s six-membered rings (Figure 5 (a)). On GOH-D, the NO_3_^–^ ion, similar to PO_4_^3–^, formed two hydrogen bonds with the hydroxyl groups (Figure 5(b)). However, in both cases the adsorption of nitrate was weaker than the adsorption of phosphate: on pristine graphene, NO_3_^–^ was adsorbed less strongly than PO_4_^3–^ by 0.89 eV. On the hydroxylated GOH-D, the difference in the adsorption energies of NO_3_^–^ and PO_4_^3–^ was even larger, at 1.36 eV. This is not caused by the higher charge of PO_4_^3–^: as seen in Figure 5(c), all phosphate ions adsorbed more strongly than NO_3_^–^, both on pristine graphene and especially on GOH-D. This consistently stronger adsorption of phosphates compared to nitrate suggests that both pure graphene and functionalized graphenes will provide selectivity of graphene-based sensors toward phosphates relative to nitrate.

### Electrical Conductivity Calculations

Graphene-based sensors typically measure the change in electrical conductivity of the sensor material as a result of adsorption of the analyte, such as gas molecules.^11−13,19^ Therefore, to investigate the sensing ability of graphene for the detection of phosphate, we calculated the electrical conductivities of pristine graphene and functionalized graphene compounds before and after adsorption of phosphate species using the Boltzmann transport equation, as described in the Computational Details section.

**Figure 6:**
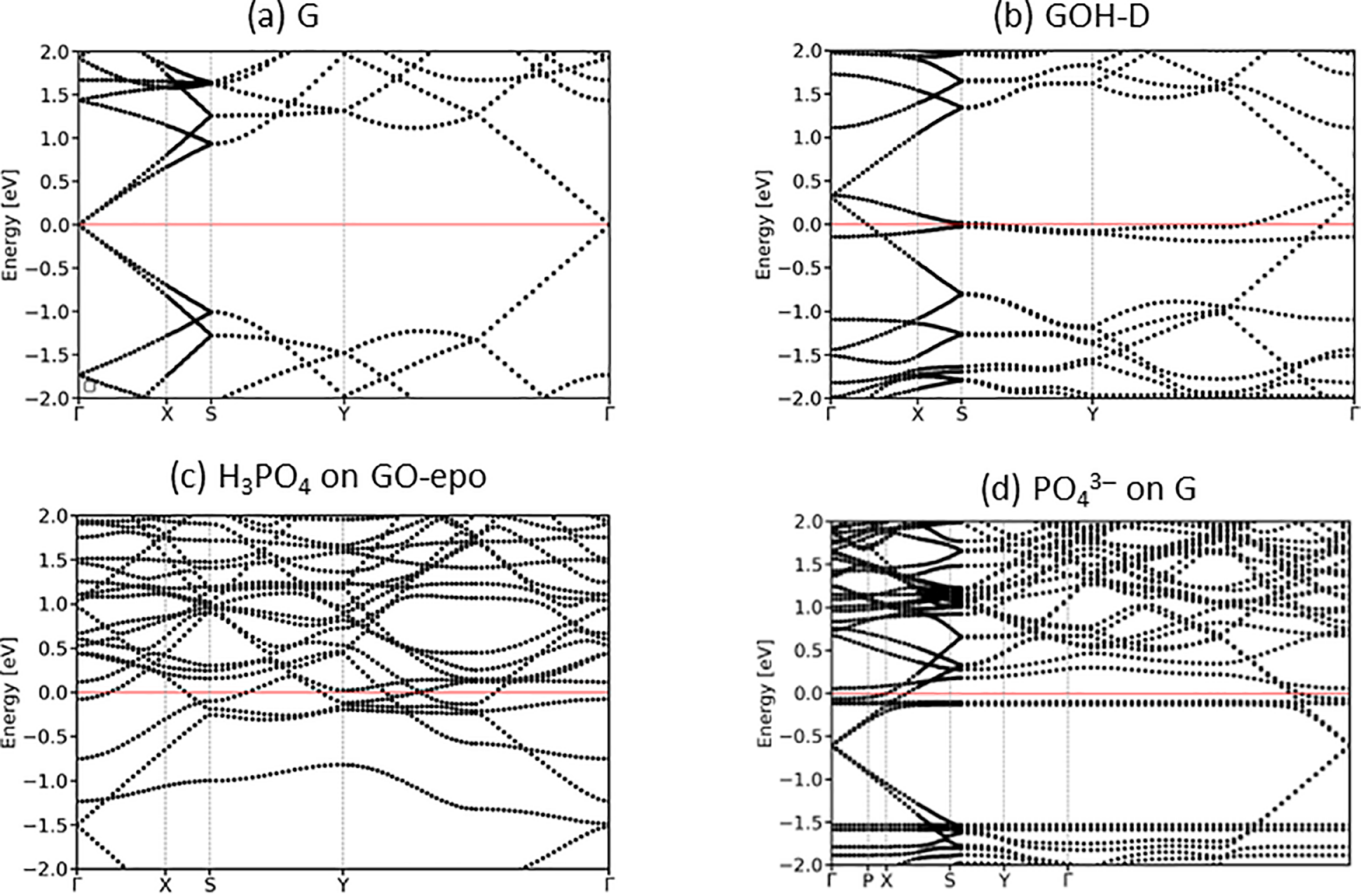
Band structures for (a) pure graphene, (b) hydroxylated structure GOH-D, (c) graphene with epoxide with adsorbed H_3_PO_4_, and (d) pure graphene with adsorbed PO_4_^3–^. The Fermi level is shown as a horizontal red line.

As the first step toward the calculations of transport properties, band structures of pristine graphene and functionalized graphene compounds with and without phosphate adsorbates were calculated. The band structure plots are presented in Figures S4–S8, and selected band structures are shown in Figure 6. Pristine graphene showed a semimetallic band structure (Figure 6(a)), with valence and conduction bands (i.e., the π and π* bands) crossing at the Dirac point. As a result of the choice of the 6 × 6 rectangular supercell model, the Dirac point appeared at the Γ point of this supercell. In the armchair and zigzag curved graphene structures, because of their reduced symmetry, the Dirac point was slightly shifted from the Γ point toward the Y and X points, respectively; armchair-GC remained semimetallic, while zig-GC appeared weakly *p*-type doped and gained some metallic character, with the π and π* bands crossing point slightly above the Fermi level. Epoxide-containing graphene and hydroxyl-containing graphene models GOH-ortho, GOH-ortho-2, and GOH-para had very similar band structures, which also resembled pristine graphene: although a new flat band appeared above the Fermi level, this did not change the semimetallic nature of the band structure, with the valence and conduction band crossing at the Fermi level. In contrast, in the hydroxylated structure GOH-D (Figure 6(b)) the flat band appeared at a lower energy and crossed the Fermi level, while the crossing point of the π and π* bands was now above the Fermi level. The vacancy-containing graphene structures also contained new flat bands (Figure S4): in the oxygen-filled vacancy (GV-O) structure, a flat band appeared at the same energy as the Dirac point and caused the shift of the Dirac point below the Fermi level. In the vacancy structure, the Dirac point was shifted slightly above the Fermi level, and the Fermi level crossed both the valence band and the new almost-flat band. Thus, while most functionalized graphenes have graphene-like band structures and are expected to have similar electronic properties, there are some exceptions: the hydroxylated structure GOH-D and the vacancy and oxygen-filled vacancy structures show a noticeable change in the band structures. These changes in the band structures can be expected to affect their transport properties.

Next, we investigated the effect of adsorption of the different phosphate species on the band structure of graphene and functionalized graphenes (Figures S5–S8). Adsorption of the neutral H_3_PO_4_ species generally had little effect on the band structures of the graphene substrates, although some bands were split into pairs of very close-lying bands as a result of symmetry breaking. The exceptions were GO-epo and GV, where the crossing point of the π and π* bands shifted significantly down (−1.5 eV in GO-epo and −1.0 eV in GV), and the Fermi level now was deep in the valence band. Adsorption of phosphate ions had a greater effect on the band structure than the adsorption of the neutral phosphoric acid, and the change was larger for the phosphate ions with larger charges: from the relatively small changes when H_2_PO_4_^–^ was adsorbed to large changes for HPO_4_^2–^ and PO_4_^3–^ adsorbates. The splitting of bands into close-lying pairs or groups of bands became more prominent when highly charged ions were adsorbed. More importantly, the adsorption of phosphate ions resulted in electron transfer and therefore in *n*-type doping of graphene, which caused a shift of the graphene Fermi level of the graphene-adsorbate systems. As a result, the Fermi level was no longer at the Dirac point in the majority of our graphene/phosphate systems, so that multiple bands crossed the Fermi level; this increased density of electronic states at the Fermi level would provide higher electrical conductivity.

**Figure 7:**
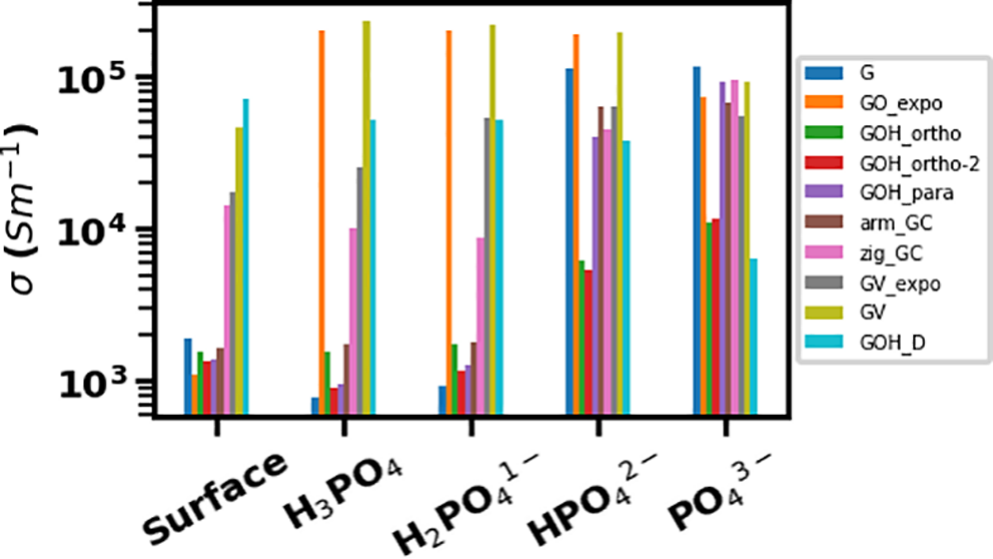
Electrical conductivities of graphene and functionalized graphene compounds before adsorption (clean surface) and after adsorption of phosphates on the functionalized graphene surfaces.

Electrical conductivities of all graphene and functionalized graphene compounds before and after adsorption of phosphate species are presented in Figure 7. Electrical transport was calculated both along the *x* and *y* directions, but the calculated transport properties were similar along both directions; therefore, we only reported the transport along the *x* direction. The leftmost part of Figure 7 shows the electrical conductivities of the graphene materials without adsorbates. It can be seen that there is a group of materials, including graphene with epoxide, all hydroxylated graphenes except GOH-D, and armchair curved graphene, which have conductivities similar or slightly lower than graphene. This is consistent with their band structures being very similar to that of pristine graphene. Only the zigzag curved graphene, graphene with a vacancy and oxygen-filled vacancy, and the hydroxylated DOH-D, whose band structures differed from graphene and showed *n*- or *p*-type doping, have significantly larger electrical conductivities.

When phosphate species were adsorbed, several different trends could be seen, depending on both the adsorbing species and the nature of the graphene material. When H_3_PO_4_ and H_2_PO_4_^–^ were adsorbed, the conductivities of several systems either stayed at the same as without adsorbate or decreased. In particular, the conductivities of pure graphene and several hydroxylated graphenes (GOH-ortho-2 and GO-para) decreased upon adsorption. Another hydroxylated graphene (GOH-ortho) and armchair curved graphene had conductivities similar to pristine graphene, which remained unchanged upon addition of the adsorbates. The hydroxylated GOH-D and zigzag curved graphene had high conductivities, which slightly decreased when the adsorbate was added. The only graphene materials whose conductivities significantly increased upon adsorption of H_3_PO_4_ and H_2_PO_4_^–^ were graphene with a vacancy and with an oxygen-filled vacancy, and especially graphene with epoxide (GO-epo), which also showed large changes in their band structures, i.e., a downshift of the Fermi level (Figure 6 (c), Figures S5 and S6). We hypothesize that this unusual behavior may be caused by the nonsymmetric charge distribution in graphene with vacancy or with epoxide and by possible repulsion between the electronegative oxygen of phosphate and the lone pair electrons at the graphene vacancy site or the epoxide oxygen.

In contrast, adsorption of HPO_4_^2–^ and PO_4_^3–^ enhanced the electrical conductivities of pure graphene and hydroxylated graphenes by up to 2 orders of magnitude. In particular, an increasing trend can be seen in the electrical conductivities of pure graphene and most of the hydroxyl-functionalized and curved graphenes, with the conductivities increasing as the charge of the adsorbed phosphate species increases. This trend would be useful in a sensor for detection of adsorbed phosphates and may be useful for identifying particular phosphate species. Graphene with epoxide, which shows high electrical conductivity when any phosphate species is adsorbed, in contrast to the low conductivity without adsorbate, is also a good candidate for a phosphate sensor material. By comparison, graphene with a vacancy or an oxygen-filled vacancy, which have high conductivities even without adsorbates, do not show large changes when phosphate species are adsorbed and therefore are not useful for a phosphate sensor. Surprisingly, the strongly adsorbing hydroxylated GOH-D structure showed the opposite trend of decreasing conductivity when PO_4_^3–^ was adsorbed. Thus, the trends in the conductivities of graphene-based materials with adsorbates are complex, and there is no universal trend. Therefore, these materials’ performance in a sensor would depend on the details of the chemical nature of the functionalized graphene material (e.g., pristine vs hydroxyl- or epoxide-containing vs vacancy-containing graphene), as well as on the nature of the phosphate species (H_3_PO_4_ and H_2_PO_4_^–^ which slightly decrease the conductivities of most materials, vs HPO_4_^2–^ and PO_4_^3–^ which lead to increased conductivities). Our calculations suggest that pure graphene and functionalized hydroxyl- and epoxide-containing and curved graphenes are good candidates for sensors because they show clear trends upon adsorption of phosphates. The prospect of using oxygen-functionalized graphene-based materials, such as graphene oxide and reduced graphene oxide, as phosphate sensors is particularly attractive, but it requires synthetic strategies to selectively produce specific arrangements of functional groups, such as hydroxyls and epoxides.

### Experimental Measurements of Phosphate–Graphene Interactions

**Figure 8:**
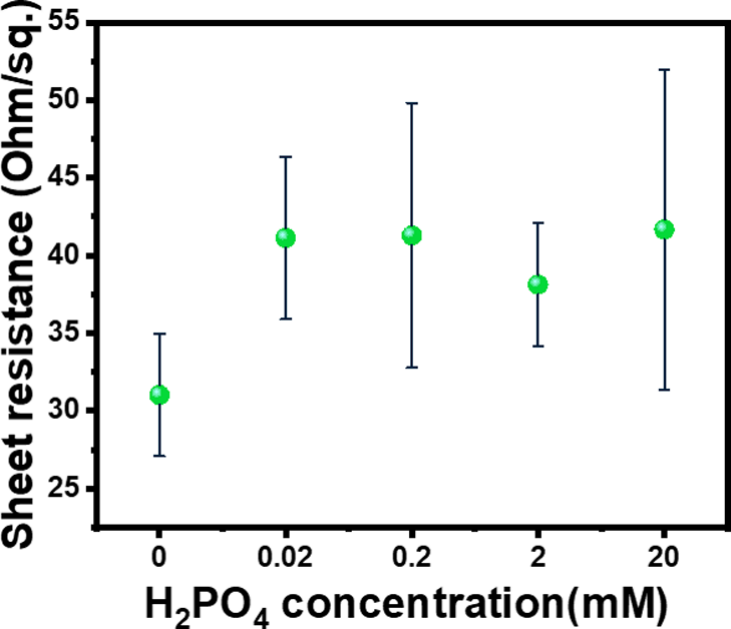
Sheet resistance of graphene samples without phosphate and with phosphate at different H_2_PO_4_^–^ concentrations.

In order to verify the change in graphene’s electrical properties as it interacts with phosphate ions and hence to validate the suitability of its use for potential development of phosphate sensor, a representative aqueous solution of phosphate ions was prepared and gently drop cast and dried on graphene surface. Then, the electrical sheet resistance of graphene with adsorbed phosphate and a bare graphene sample was measured, as described in the Experimental Details section. Dihydrogen phosphate was used as a representative phosphate species, because it is the dominant species in the pH range 2.2–7.2, which is in the typical soil pH range.^9^ The protonation state of dihydrogen phosphate was maintained at the solution pH between 4 and 6. As seen in Figure 8, the sheet resistance increased as the phosphate was added to the graphene surface compared to the bare graphene sample. This change in the conductivity is larger than the uncertainty indicated by error bars, confirming a robust trend in the conductivity change upon addition of phosphate. However, there was no clear trend as a function of the concentration of phosphate in the droplets, with the sheet resistance variation of all phosphate-containing samples being smaller than the error bars.

The key result showing an increase in sheet resistance upon the adsorption of phosphate is consistent with the calculated conductivity trends for pure graphene (blue bars in Figure 7). The calculations presented in the previous section showed a decrease in the conductivity of pure graphene (and therefore an increase in resistivity) upon adsorption of H_3_PO_4_ and H_2_PO_4_^–^. Thus, the representative experimental result confirms the computational prediction. Furthermore, following the measurements of this model system, phosphate binding to graphene and its impact on electrical conductivity could be used to assess other engineered graphenes proposed in this work as materials for phosphate sensors.

## Discussion

Our aim was to assess the usability of graphene and functionalized graphenes as sensing materials in chemoresistive sensors, which measure the resistance of a sensing layer and detect a change in resistance of the sensing layer after adsorption of the analyte species.^49,50^ Our calculations showed that adsorption of phosphates affected the electronic properties of graphene (as illustrated in the band structure plots in Figure 6 and Figures S5–S8), which resulted in a change in graphene’s electrical conductivity. In particular, adsorption of phosphate ions resulted in electron transfer from adsorbates to graphene and therefore in *n*-type doping of graphene, which increased the conductivity (and decreased the resistivity) of graphene substrates in many of the phosphate/graphene pairings considered in our theoretical modeling. In some instances with H_3_PO_4_ and H_2_PO_4_^–^ adsorbates, the opposite effect was seen in the calculations, where the conductivity decreased, which can be attributed to a decreased carrier mobility. Thus, our calculations predicted that the adsorption of phosphate species would cause a measurable electronic response, which was validated by experimental measurements of electrical resistance of graphene samples with and without adsorbates.

The calculated adsorption energies spanned a broad range, from −0.18 to −0.87 eV for the neutral phosphoric acid up to −2.25 to −3.23 eV for the PO_4_^3–^ ion. Thus, it is clear that the strength of adsorption differs for the different phosphate species, with stronger adsorption for the more highly charged species. The charge transfer data shown in Figure 4 explain the reason for this trend: larger charge transfer is observed from the highly charged PO_4_^3–^ ion to graphene, compared to the hydrogenated phosphate ions with smaller charges and the neutral molecule. We interpret this large charge transfer as the reason for the stronger adsorption of the highly charged ions. These large adsorption energies and the difference between differently charged adsorbates are expected to have implications for phosphate sensor performance: the neutral molecule with relatively weak adsorption should be able to adsorb and desorb reversibly, thus making a sensor reusable. In contrast, the PO_4_^3–^ ion adsorbs strongly and its desorption is not favorable. This is likely to result in the sensor surface becoming fully covered with adsorbate over time. This would limit the lifetime of a sensor and reduce its sensitivity over time, as less of the graphene surface would be exposed, therefore making the sensor not reusable. Since the nature of the dominant phosphate species in aqueous solution depends on the pH of the solution,^8^ the amount of phosphate species that are strongly and irreversibly adsorbed on the sensor surface is expected to depend on the pH, with large amounts of strongly adsorbed PO_4_^3–^ at low pH and mostly weakly adsorbed H_3_PO_4_ at high pH. At pH values relevant to soil (pH 4.5–7.8),^9^ the dominant species are H_2_PO_4_^–^ and HPO_4_^–^ which adsorb quite strongly (−1.40 to −2.97 eV) and are likely to fully cover the sensor surface over time. Therefore, such sensors will necessarily have limited lifetimes, and their sensitivity will decrease over time.

Our findings on the performance of graphene-based sensors have implications beyond phosphate sensing. While the research field of gas sensing is already well developed,^11−13,17,19^ this study opens the possibility of electrical sensing of ions in solution, using cheap disposable graphene-based resistance sensors. This approach can be applied to other soil nutrients, such as nitrate and potassium, and to detect pollutant species in water. Beyond the field of sensors, the strong binding ability of functionalized graphenes suggest that such ions can be used for environmental remediation, to remove pollutants such as heavy metal from water and soil.^27^

There are limitations to this study: in particular, the calculations considered only a single adsorbate interacting with the graphene surface and did not consider the solvent environment and presence of counterions. As discussed above, the presence of solvent affects adsorption enthalpies; inclusion of explicit solvent would give a clearer picture of adsorption and desorption of phosphate and entropic effects. Moreover, selectivity was not fully addressed: competing adsorbed species, such as nitrate, are likely to produce a similar electrical response in graphene, but deconvoluting the effects of competing adsorbed species is beyond our theoretical capabilities.

In the experimental measurements, potential sources of errors could arise from (1) slight variation in the drop size and evenness of phosphate distribution over the graphene drop, (2) the surface cleanliness of the polyimide substrates on which the graphene ink is coated, and (3) drying conditions of graphene ink. However, we have minimized all these variations to our best ability with careful experimentation and by taking measurements on multiple samples.

## Conclusions

We investigated graphene and functionalized graphene-based nanomaterials (oxygenated graphene, curved graphene, and graphene with a vacancy) as potential sensor materials for the detection of phosphates in the environment, such as water and soil. Our DFT calculations showed that phosphate, monohydrogen phosphate, and dihydrogen phosphate ions (PO_4_^3–^, HPO_4_^2–^, H_2_PO_4_^–^) adsorbed strongly as a result of electrostatic interactions, surface–adsorbate charge transfer, and hydrogen bonding. All phosphate species adsorbed more strongly than nitrate, which is also commonly found in soil and can be seen as a competing species. Phosphoric acid H_3_PO_4_ adsorbed the least strongly, primarily through dispersion interactions and hydrogen bonding. Several types of engineered graphenes, such as graphene containing hydroxyl groups, graphene with vacancies, and curved graphene showed stronger adsorption for all the phosphate species compared with pristine graphene. This strong adsorption suggested functionalized graphenes as promising candidates for detection of phosphate. Calculations of electrical conductivities of these functionalized graphene nanomaterials with and without phosphate showed significant changes in conductivities after adsorption of phosphate species, suggesting that these graphene nanomaterials are promising electrical sensor materials for detection of phosphate. These predictions were validated by proof-of-concept electrical resistance measurements of a representative system, pure graphene without and with adsorbed dihydrogen phosphate H_2_PO_4_^–^. These results support graphene as a suitable sensor material for the detection of phosphate in water and soil. Furthermore, based on our theoretical predictions, we anticipate that suitably designed hydroxyl-functionalized graphene would be ideal sensor materials for charged phosphates. Such sensors would enable achieving the overall goal of developing easy-to-use phosphate sensors for sustainable agriculture.

## Supporting Information Available

Images of adsorption configurations of HPO_4_^2–^, H_2_PO_4_^–^, and H_3_PO_4_; tables of adsorption energies; band structures of the engineered graphene materials with and without adsorbates (PDF)
