# Supplementary material for: Theoretical and Experimental Studies of Molecular Interactions between Engineered Graphene and Phosphate Ions for Graphene-Based Phosphate Sensing
Source: ACS Appl Nano Mater. 2023 Dec 18;7(16):18386–97. doi: 10.1021/acsanm.3c04147 (PMC11348312; doi:10.1021/acsanm.3c04147)
Supplement: Supplementary file 1 — an3c04147_si_001.pdf [file an3c04147_si_001.pdf]

Supporting Information for

Theoretical and Experimental Studies of Molecular Interactions Between Engineered Graphene and Phosphate Ions for Graphene-Based Phosphate Sensing

Xue Yong,[a] Thiba Nagaraja,[b] Rajavel Krishnamoorthy,[b] Ana Guanes,[b] Suprem R. Das[b,c] and Natalia Martsinovich[a]*

[a] Department of Chemistry, University of Sheffield, Sheffield S3 7HF, United Kingdom

[b] Department of Industrial and Manufacturing Systems Engineering, Kansas State University, Manhattan, Kansas, 66506, United States

[c] Department of Electrical and Computer Engineering, Kansas State University, Manhattan, Kansas, 66506, United States

*Corresponding author: n.martsinovich@sheffield.ac.uk

Figure S1. The most stable adsorption configuration for HPO_4^{2-} on pristine graphene (G), hydroxyl- (GOH) and epoxide-containing (GO-epo) graphene, curved graphene (arm-GC and zig-GC), graphene with vacancy (GV) and with oxygen-filled vacancy (GV-O). Top views are shown for all systems, except for arm-GC and zig-GC where side views are shown. In all figures, carbon atoms are shown in brown, oxygen atoms in red, phosphorus atoms in light purple, and hydrogen atoms in white.

Figure S2. The most stable adsorption configuration for $H_2PO_4^-$ on pristine graphene (G), hydroxyl- (GOH) and epoxide-containing (GO-epo) graphene, curved graphene (arm-GC and zig-GC), graphene with vacancy (GV) and with oxygen-filled vacancy (GV-O). Top views are shown for all systems, except for arm-GC and zig-GC where side views are shown.

Figure S3. The most stable adsorption configuration for H_3PO_4 on pristine graphene (G), hydroxyl- (GOH) and epoxide-containing (GO-epo) graphene, curved graphene (arm-GC and zig-GC), graphene with vacancy (GV) and with oxygen-filled vacancy (GV-O). Top views are shown for all systems, except for arm-GC and zig-GC where side views are shown. In all figures, carbon atoms are shown in brown, oxygen atoms in red, phosphorus atoms in light purple, and hydrogen atoms in white.

Table S1. Adsorption energies of phosphate species on graphene-based materials in vacuum.

Table S2. Adsorption energies of phosphate species on graphene-based materials in implicit water solvent.

Figure S4. Band structures for pure graphene and modified graphenes without adsorbate. The band structure for GOH-ortho-2 is not shown in this figure and the following figures, because it is very similar to GOH-ortho.

Figure S5. Band structures for pure graphene and modified graphenes with H_3PO_4 adsorbate.

Figure S6. Band structures for pure graphene and modified graphenes with $H_2PO_4^-$ adsorbate.

Figure S7. Band structures for pure graphene and modified graphenes with HPO_4^{2-} adsorbate.

Figure S8. Band structures for pure graphene and modified graphenes with PO_4^{3-} adsorbate.

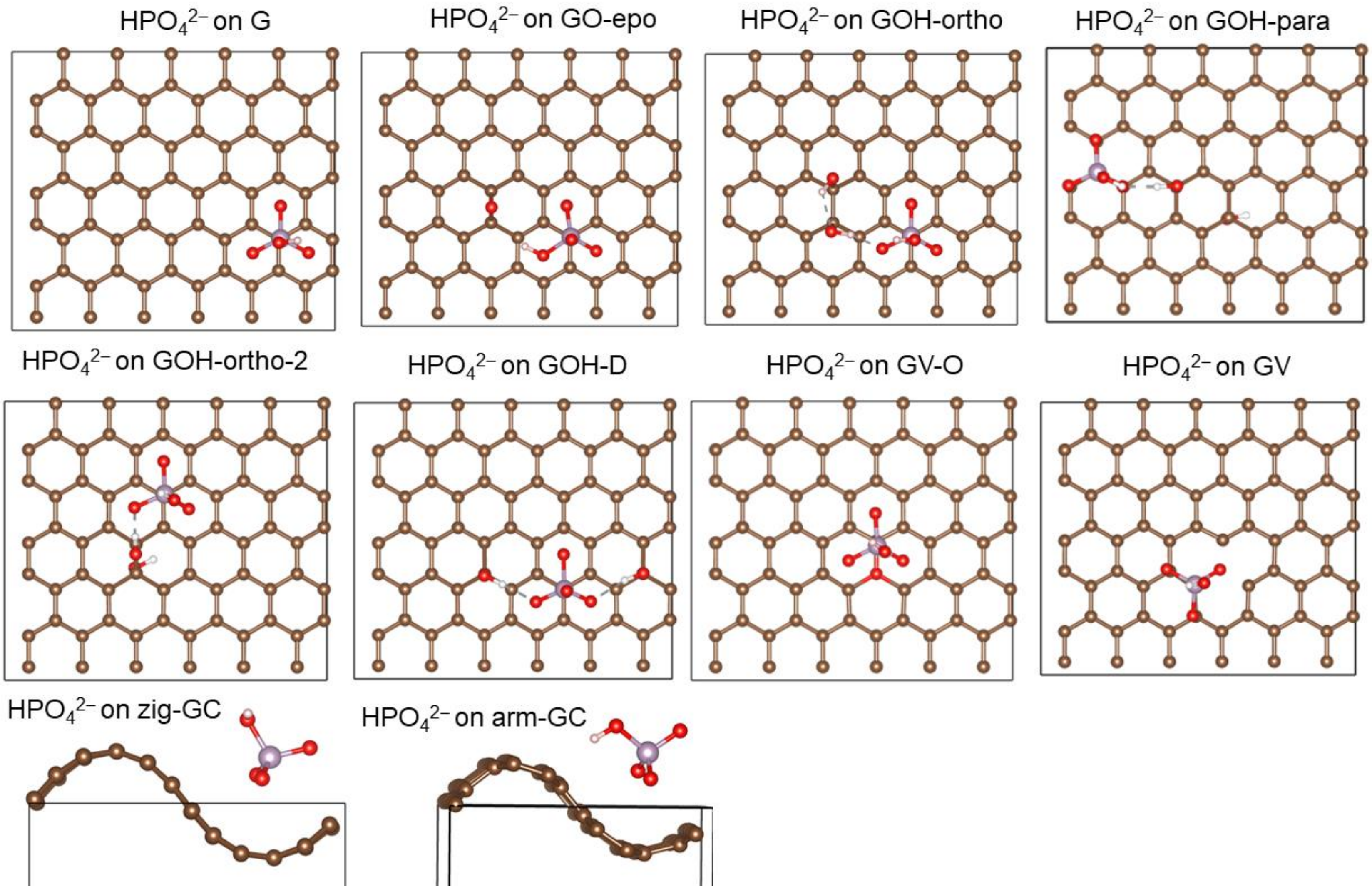

Figure S1. The most stable adsorption configuration for HPO_4^{2-} on pristine graphene (G), hydroxyl- (GOH) and epoxide-containing (GO-epo) graphene, curved graphene (arm-GC and zig-GC), graphene with vacancy (GV) and with oxygen-filled vacancy (GV-O). Top views are shown for all systems, except for arm-GC and zig-GC where side views are shown. In all figures, carbon atoms are shown in brown, oxygen atoms in red, phosphorus atoms in light purple, and hydrogen atoms in white.

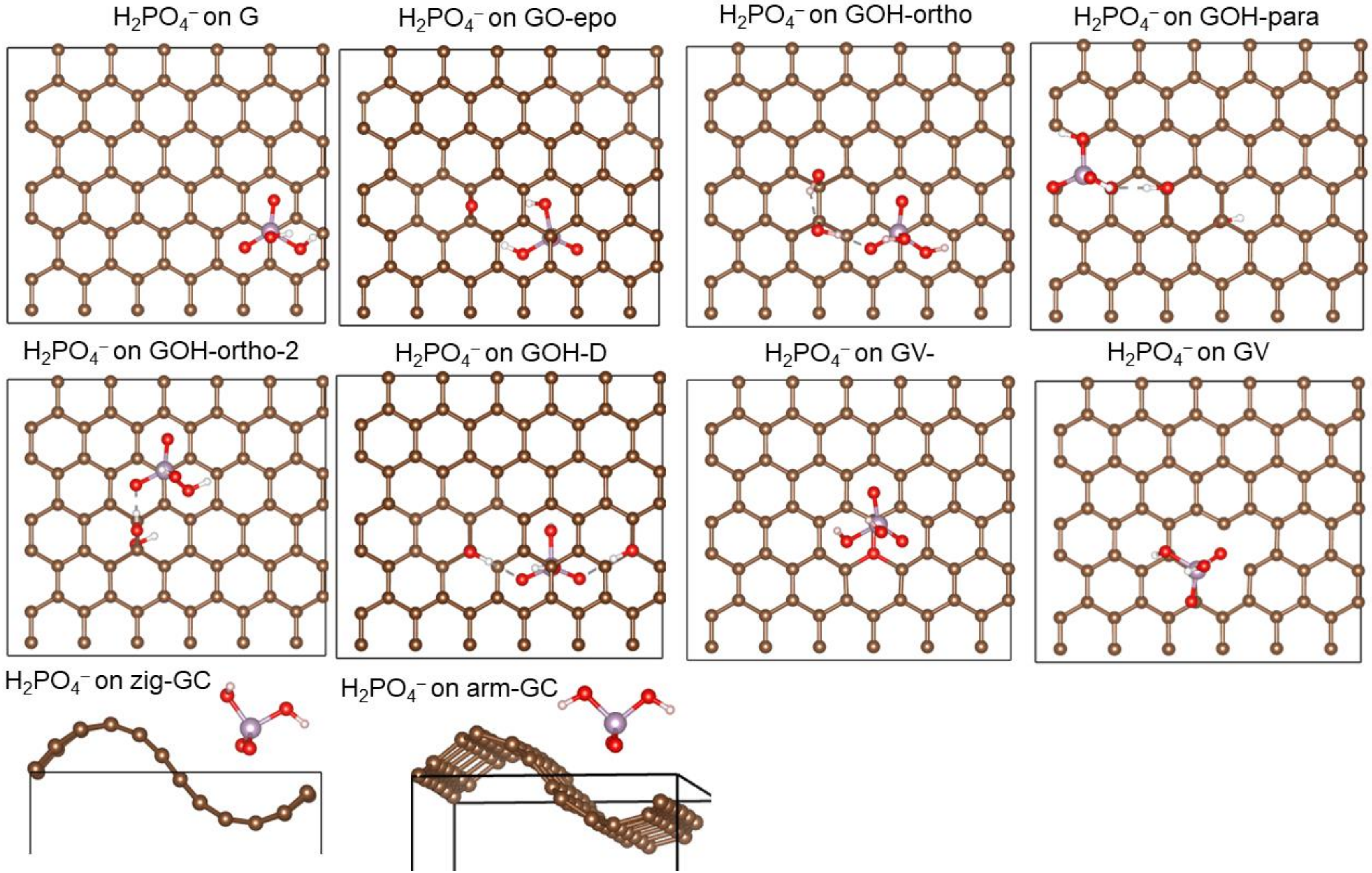

Figure S2. The most stable adsorption configuration for $H_2PO_4^-$ on pristine graphene (G), hydroxyl- (GOH) and epoxide-containing (GO-epo) graphene, curved graphene (arm-GC and zig-GC), graphene with vacancy (GV) and with oxygen-filled vacancy (GV-O). Top views are shown for all systems, except for arm-GC and zig-GC where side views are shown.

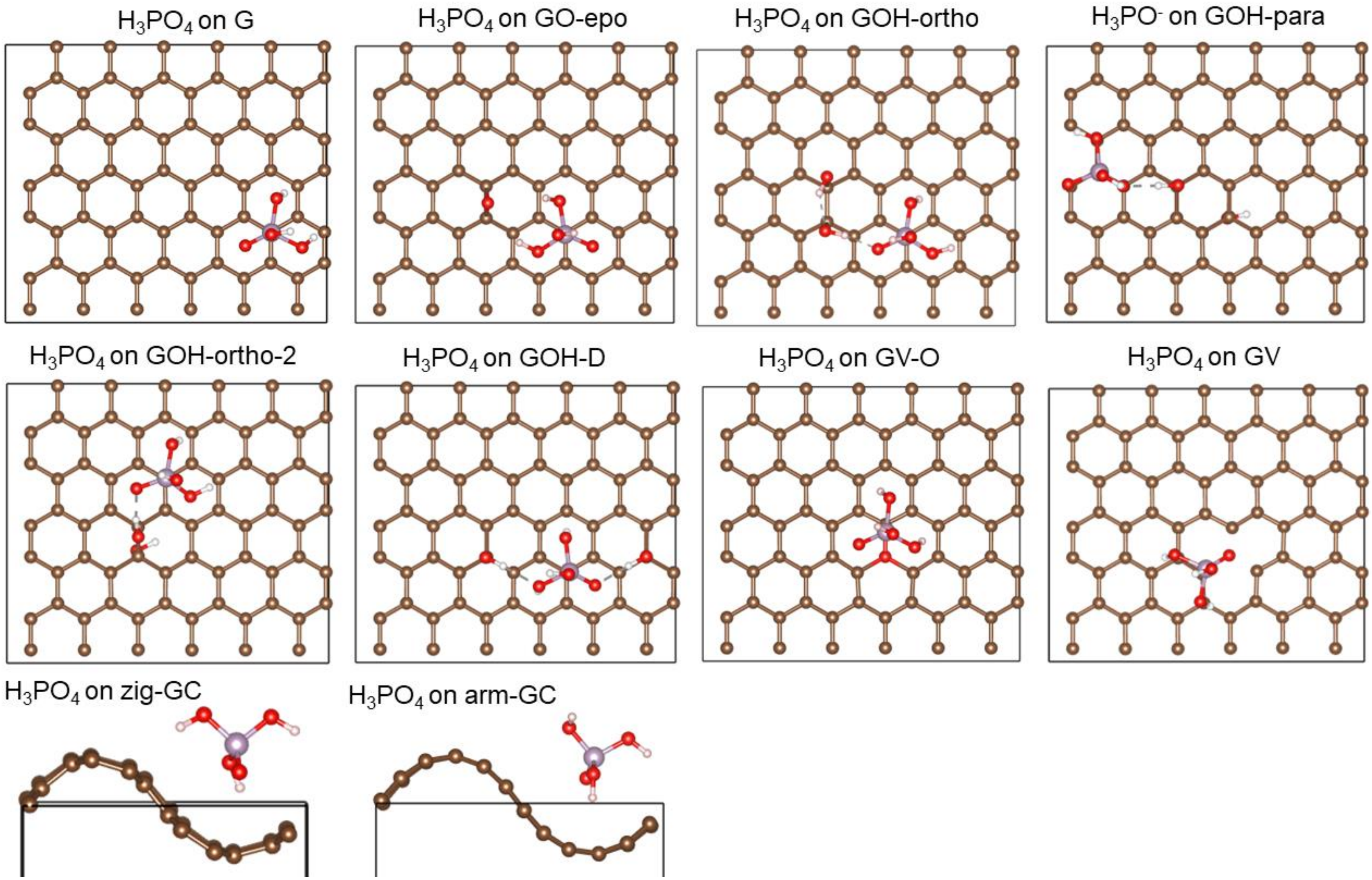

Figure S3. The most stable adsorption configuration for H_3PO_4 on pristine graphene (G), hydroxyl- (GOH) and epoxide-containing (GO-epo) graphene, curved graphene (arm-GC and zig-GC), graphene with vacancy (GV) and with oxygen-filled vacancy (GV-O). Top views are shown for all systems, except for arm-GC and zig-GC where side views are shown.

Table S1. Adsorption energies of phosphate species on graphene-based materials in vacuum.

	Adsorbates			
**Graphene materials**	**PO_4^{3-}**	**HPO_4^{2-}**	**$H_2PO_4^{-}$**	**H_3PO_4**
G	-2.28	-2.14	-1.63	-0.18
GO-epo	-2.25	-1.95	-1.40	-0.54
GOH-ortho	-2.85	-2.73	-2.41	-0.87
GOH-ortho-2	-2.72	-2.50	-2.15	-0.53
GOH-para	-2.69	-2.50	-2.20	-0.63
GOH-D	-3.23	-2.57	-2.54	-0.66
zig-GC	-3.04	-2.74	-2.26	-0.60
arm-GC	-2.86	-2.61	-2.13	-0.52
GV-O	-3.19	-2.97	-2.25	-0.37
GV	-2.77	-2.42	-1.91	-0.50

Table S2. Adsorption energies of phosphate species on graphene-based materials in implicit water solvent.

	Adsorbates			
**Graphene materials**	**PO_4^{3-}**	**HPO_4^{2-}**	**$H_2PO_4^{-}$**	**H_3PO_4**
G	-3.11	-2.61	-1.73	0.15
GO-epo	-2.95	-2.28	-1.37	-0.15
GOH-ortho	-3.44	-2.99	-2.31	-0.43
GOH-para	-3.49	-2.79	-2.10	-0.21
zig-GC	-3.86	-3.04	-2.11	-0.07
arm-GC	-3.66	-2.95	-1.98	0.12
GV-O	-4.20	-3.56	-2.29	0.06
GV	-3.66	-2.97	-1.99	-0.11

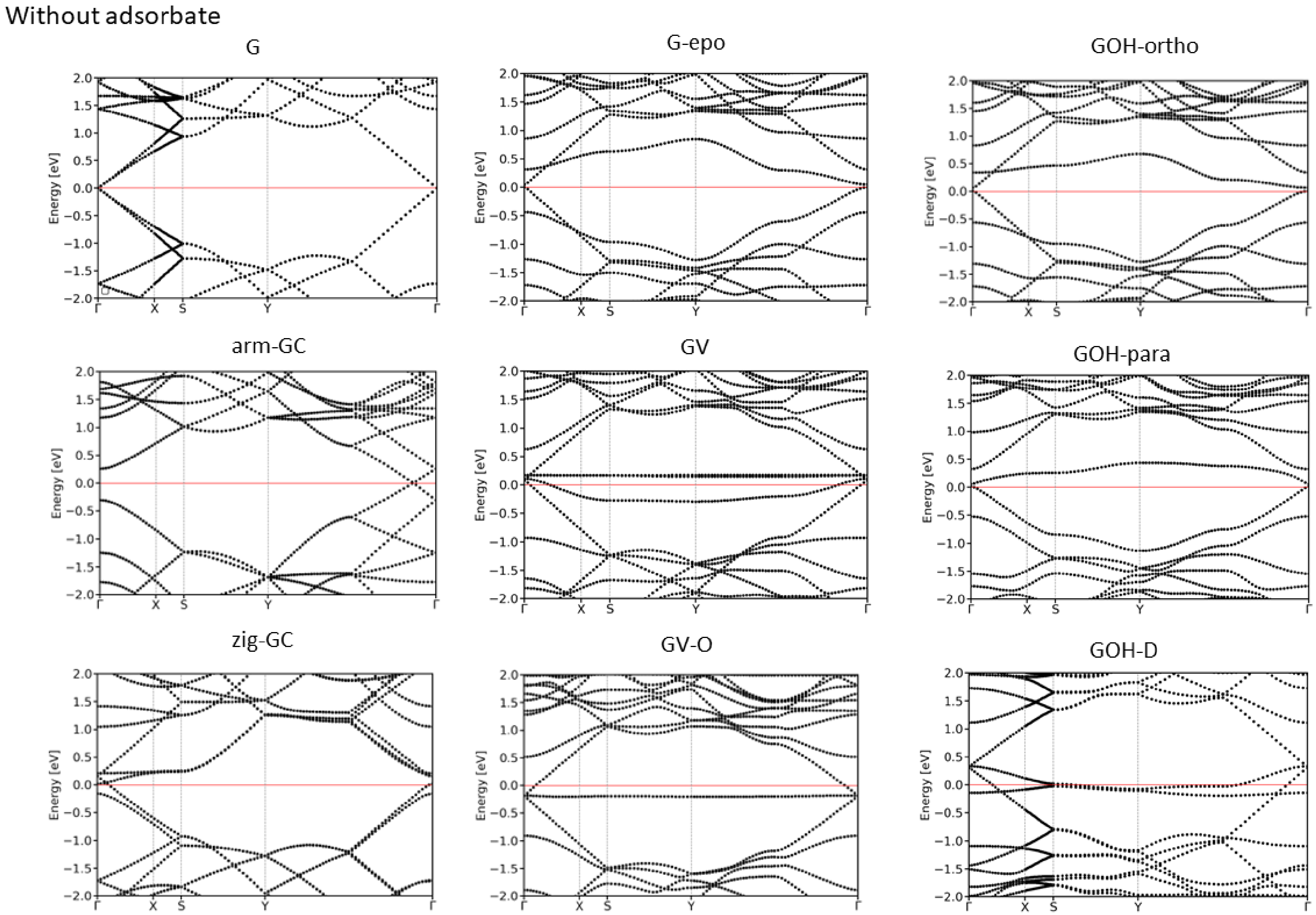

Figure S4. Band structures for pure graphene and modified graphenes without adsorbate. The band structure for GOH-ortho-2 is not shown in this figure and the following figures, because it is very similar to GOH-ortho.

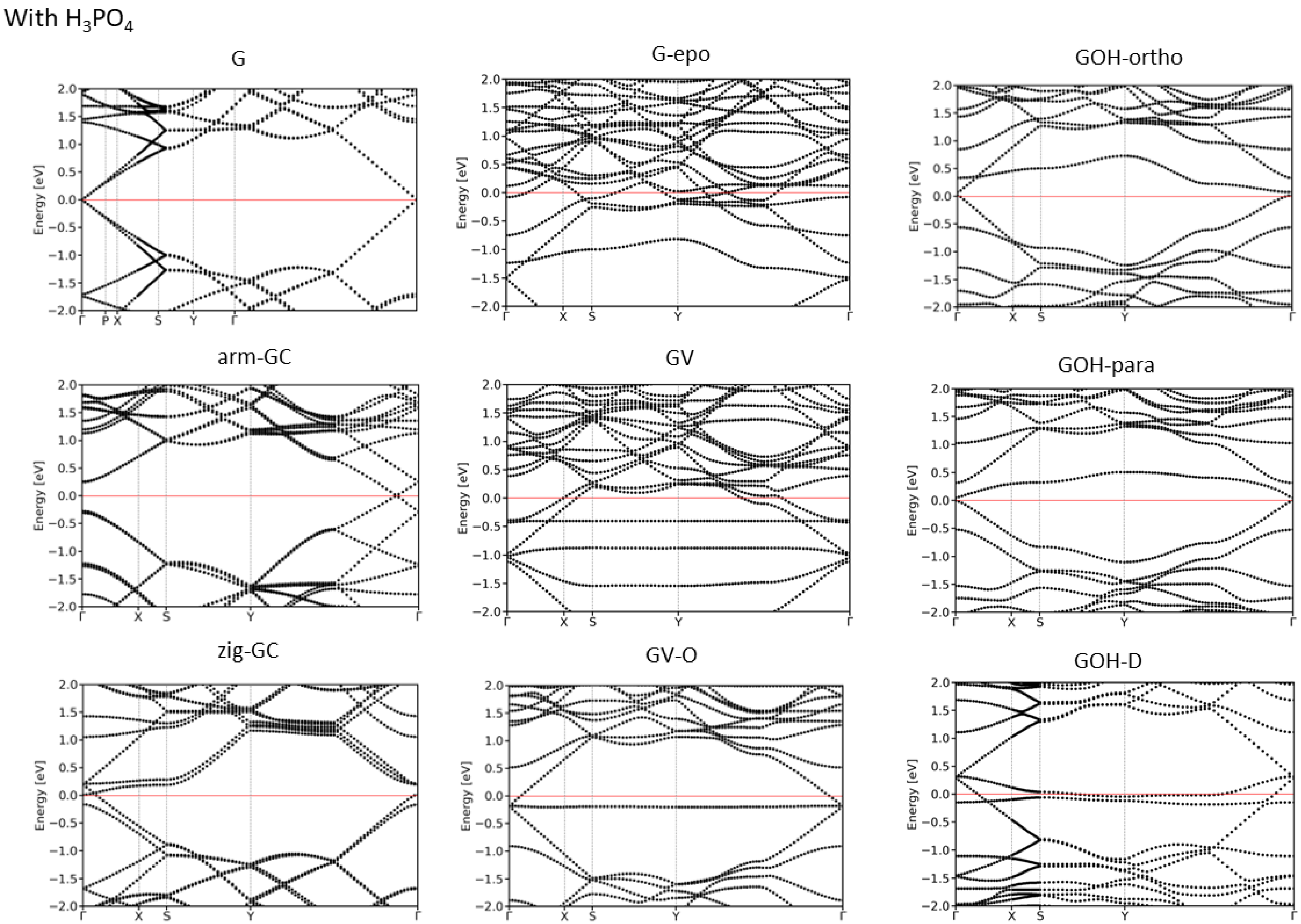

Figure S5. Band structures for pure graphene and modified graphenes with H_3PO_4 adsorbate.

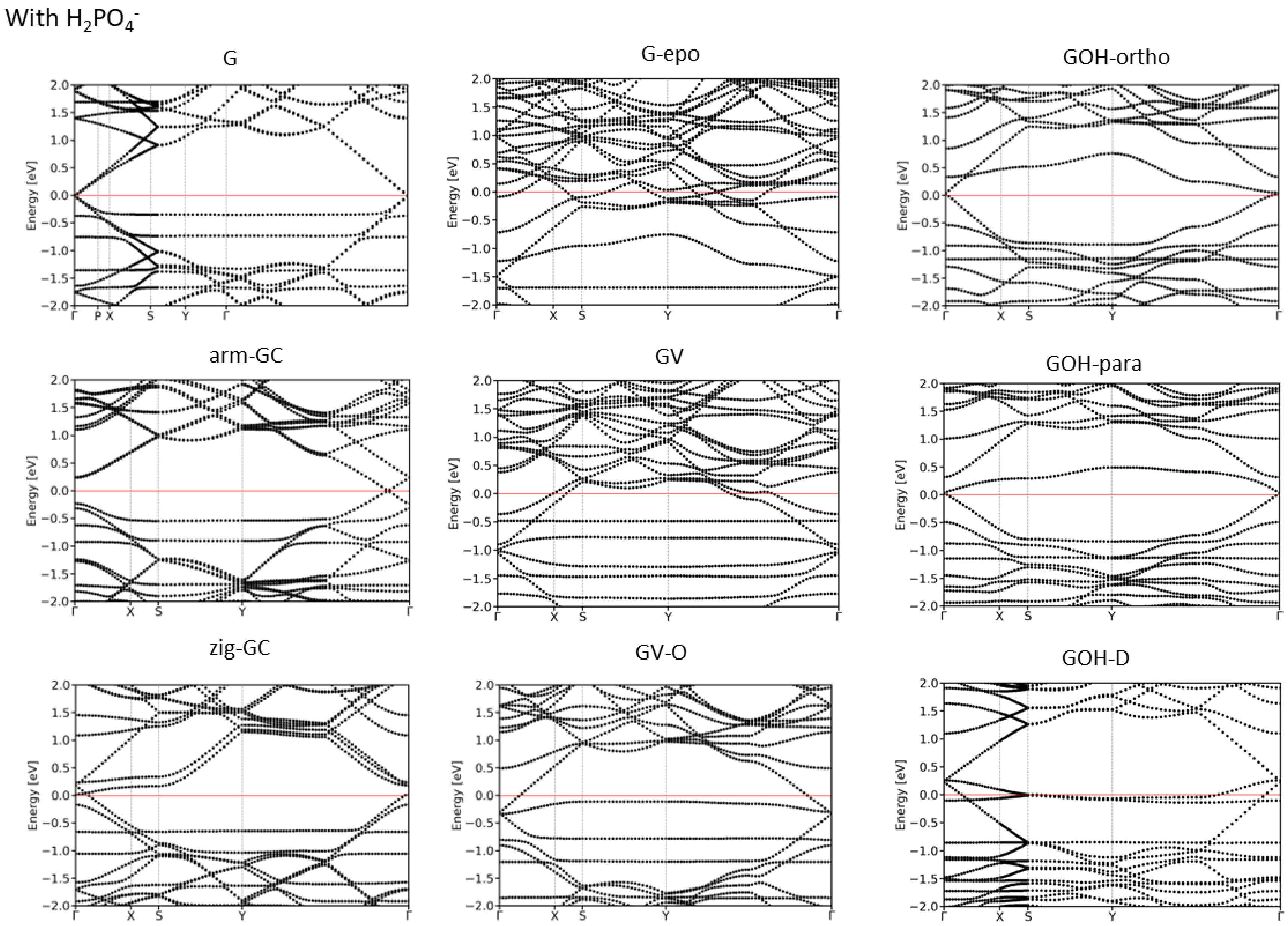

Figure S6. Band structures for pure graphene and modified graphenes with $H_2PO_4^-$ adsorbate.

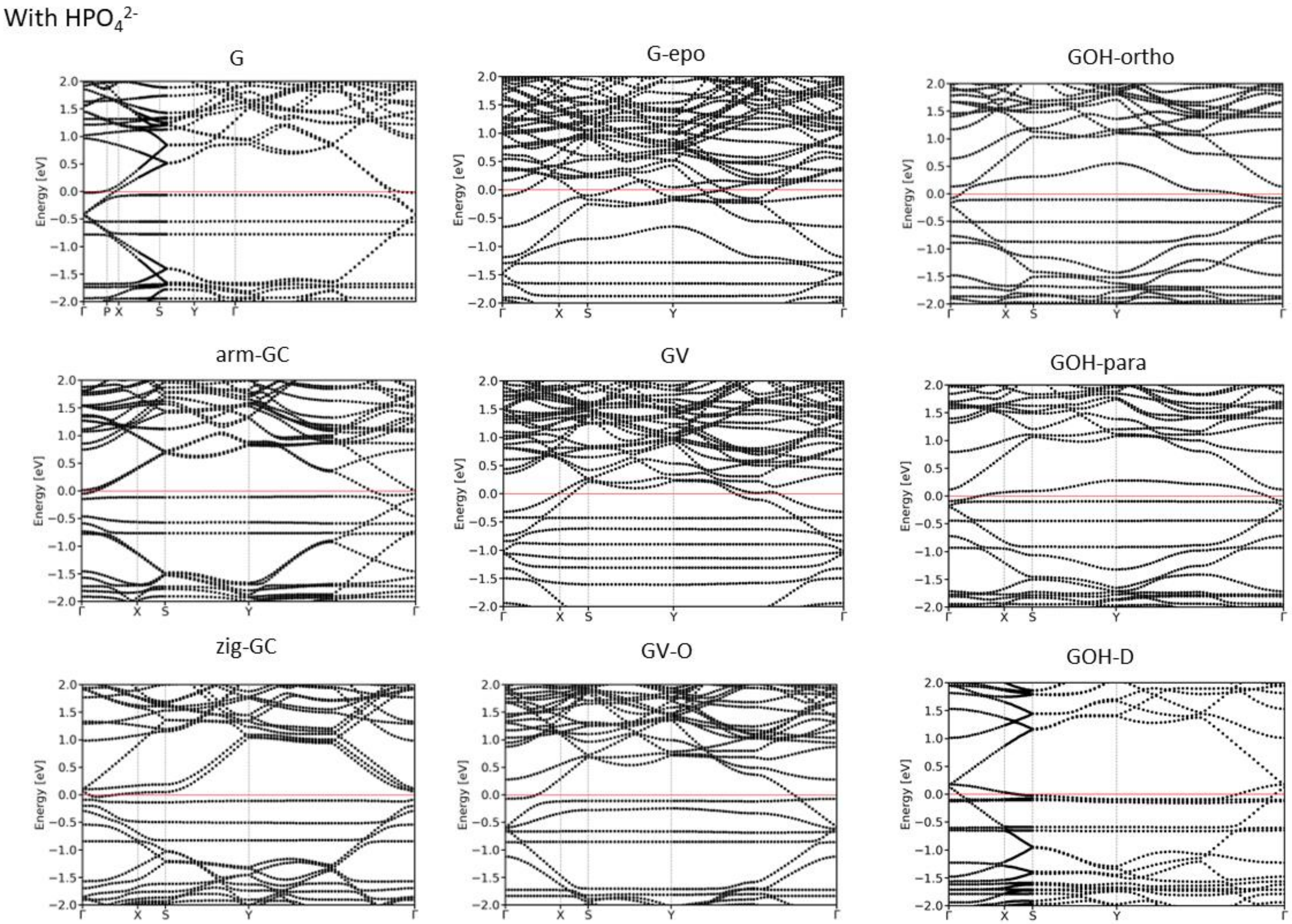

Figure S7. Band structures for pure graphene and modified graphenes with HPO_4^{2-} adsorbate.

With PO_4^{3-}

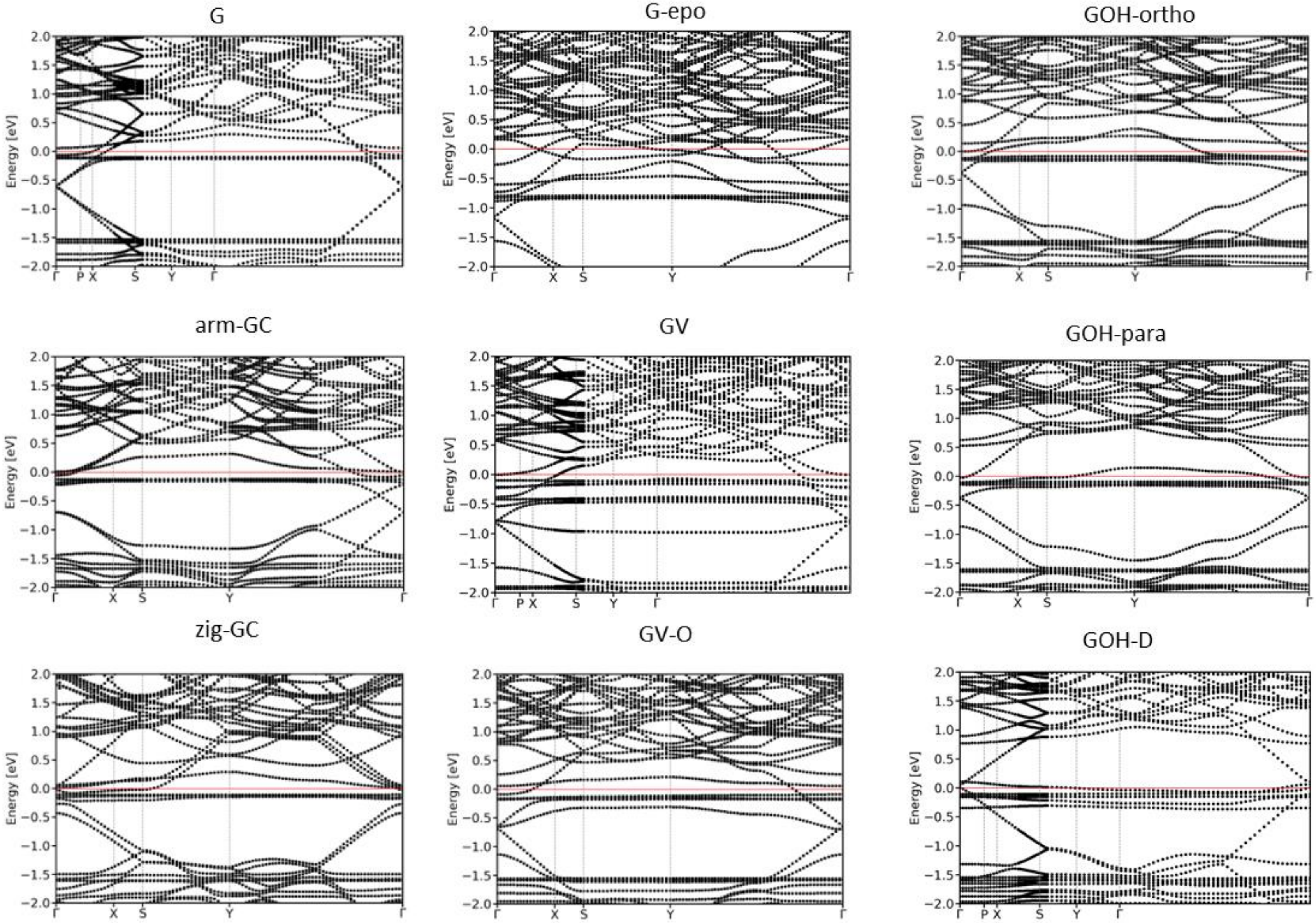

Figure S8. Band structures for pure graphene and modified graphenes with PO_4^{3-} adsorbate.
